# Recruitment, Engagement, Experiences, and Self-Reported Changes in a Digital Patient Activation Intervention to Improve Hemodialysis Session Stability: Mixed Methods Study

**DOI:** 10.2196/93049

**Published:** 2026-10-16

**Authors:** Megan Wickens, Jennifer Bragg-Gresham, Rajiv Saran, Meera Tandan, Marissa Argentina, Dinesh Chatoth, Kelli Collins Damron, Michael Heung, Sarah Krein, Vu Le, Ana Laura Licon, Kara Eggebrecht, Barbara Murphy, Alicia Zerkle, Kai Zheng, Tiffany C Veinot

**Affiliations:** 1 School of Information University of Michigan Ann Arbor, MI United States; 2 Department of Internal Medicine, Division of Nephrology School of Medicine University of Michigan Ann Arbor, MI United States; 3 Kidney Epidemiology and Cost Center University of Michigan Ann Arbor, MI United States; 4 National Kidney Foundation New York, NY United States; 5 Fresenius Medical Care (United States) Waltham, MA United States; 6 Center for Clinical Management Research Veterans Affairs Ann Arbor Ann Arbor, MI United States; 7 Department of Internal Medicine, Division of General Medicine School of Medicine University of Michigan Ann Arbor, MI United States; 8 Institute for Clinical and Translational Science University of California, Irvine Irvine, CA United States; 9 School of Information and Computer Sciences University of California, Irvine Irvine, CA United States; 10 Department of Learning Health Sciences School of Medicine University of Michigan Ann Arbor, MI United States; 11 Department of Health Behavior and Health Equity School of Public Health University of Michigan Ann Arbor, MI United States

**Keywords:** consumer health informatics, peer mentoring, patient education, intervention-generated inequality, digital inclusion, digital health equity, patient safety, patient involvement, self-efficacy, self-determination, uptake, engagement

## Abstract

**Background:**

When chronic kidney disease progresses to end-stage kidney disease, many patients receive in-center hemodialysis. Although lifesaving, hemodialysis is associated with intradialytic hypotension. Peer mentoring and patient activation support in hemodialysis have not been well studied.

**Objective:**

This Dialysafe study combined patient education and peer mentoring to promote health behaviors supporting prevention of intradialytic hypotension. We aimed to describe recruitment, engagement, completion, and attrition; assess participants’ health goals and self-reported behavior changes; and evaluate satisfaction and recommendations for improvement.

**Methods:**

At 10 Dialysafe hemodialysis centers assigned to patient activation, all eligible English-speaking patients were invited. The intervention comprised 5 educational modules and 5 videoconference peer-mentoring sessions. Mixed methods analyses integrated electronic health record and platform data, postintervention surveys, and clinic operations committee notes. An engagement score (range 0-50) summarized intervention use. Descriptive statistics, *t* tests, chi-square tests, logistic regression, and zero-inflated Poisson regression were used to assess recruitment, completion (all 5 mentoring sessions), and engagement. Qualitative data were inductively coded and integrated through joint displays.

**Results:**

Of the 639 eligible patients, 155 (24.3%) were recruited. Recruited patients were younger than nonrecruited patients (mean 57.6, SD 13.8, vs 64.3, SD 14.7 y; *P*<.001); each additional year was associated with 4% lower odds of recruitment (odds ratio [OR] 0.96, 95% CI 0.94-0.98; *P*<.001). Recruitment was more likely among college graduates (OR 1.70, 95% CI 1.02-2.83; *P*=.04) and patients with sleep disorders (OR 2.27, 95% CI 1.06-4.87; *P*=.03). Attrition was concentrated before mentoring sessions began. Of recruited patients, 72 of 155 (46.5%) met with a peer mentor and 58 of 155 (37.4%) completed all 5 sessions. Completers were 1.9 years younger (56.4, SD 13.8, vs 58.3, SD 13.9 y) and had Charlson Comorbidity Index scores 0.4 points higher (3.4, SD 2.0, vs 3.0, SD 2.3) than noncompleters. The average engagement score was 18.4 of 50 (SD 20.1). Engagement was higher among men (incidence rate ratio [IRR] 1.33, 95% CI 1.22-1.45; *P*<.001), Black patients (IRR 1.27, 95% CI 1.16-1.39; *P*<.001), and patients with dialysis vintage <1 year (IRR 1.22, 95% CI 1.05-1.42; *P*=.01). Higher Charlson Comorbidity Index scores predicted engagement (IRR 1.04, 95% CI 1.01-1.07; *P*=.002) and completion (OR 1.37, 95% CI 1.05-1.79; *P*=.02). Of 79 patients who set goals, 64 (81%) selected communication with the care team; 22 of 54 (40.7%) survey respondents identified this as their main behavior change. Both mentees and mentors identified peer-mentoring sessions as the most valuable component. Among mentee respondents, 55 of 55 (100%) respondents rated the program good or excellent, and 53 of 54 (98.1%) respondents would recommend it.

**Conclusions:**

Participants constituted a self-selecting group. Patient-clinician communication was a priority, and participants valued peer mentoring. Digital patient activation intervention strategies to increase reach are needed.

**Trial Registration:**

ClinicalTrials.gov NCT03171545; https://clinicaltrials.gov/ct2/show/NCT03171545

**International Registered Report Identifier (IRRID):**

RR2-10.2196/46187

## Introduction

### Background

Chronic kidney disease (CKD) is characterized by reduced kidney function and/or evidence of kidney damage. In the United States, 14% of adults have CKD [1]. Advanced CKD (stage 5) is called end-stage kidney disease (ESKD) when kidney replacement therapy becomes essential. Patients with ESKD require a kidney transplant or dialysis therapy; in-center hemodialysis is the most commonly used treatment modality in the United States [2].

Rapid fluid removal can lead to unstable hemodialysis sessions [3-5]. The most frequent type of hemodialysis session instability is intradialytic hypotension (IDH), or low blood pressure during hemodialysis [6]. IDH occurs in 20% of hemodialysis sessions [7]. Reducing IDH rates is a high priority in hemodialysis care, particularly given that IDH is associated with unpleasant symptoms, such as cramping, fainting, vomiting, and fatigue [8]; reduced quality of life [9]; cardiovascular disease [5,10,11]; hospitalization [12]; and mortality [13].

The risk of developing IDH can be affected by fluid-related decisions of both clinicians and patients. Key fluid-related decisions for patients include attending scheduled dialysis sessions, agreeing to extra or longer sessions, and following sodium restrictions between sessions [14]. Additional fluid-related decisions for patients comprise working with their providers to determine their optimum postdialysis target weight and their “goal” for the amount of fluid removed per session [15], as well as informing their providers about any negative symptoms during sessions. Patients may also have difficulty participating in their care due to their comprehension of fluid management, challenges with symptom recognition, role uncertainties, and understanding of communication processes [15-20]. Yet, few interventions have focused on helping patients participate actively in preventing IDH [21-23].

### Digital and Peer-Based Interventions for Chronic Disease

Outside of an IDH-specific context, digital interventions have been used to promote chronic disease management among dialysis patients [24,25] and others with chronic conditions [26]. Often, the goal of interventions for patients with chronic conditions, such as ESKD, includes enhancing patient activation [27], or “an individual’s knowledge, skill, and confidence for managing their health and health care” [28].

Among patient activation intervention approaches, patient peer support or mentoring may be valuable for complex chronic illnesses, such as ESKD [29]. Peer support is defined as “...the provision of emotional, appraisal, and informational assistance by a created social network member who possesses experiential knowledge of a specific behavior or stressor and similar characteristics as the target population” [30]. Peer support is less structured or behavior-change-focused than peer mentoring, which we define as “...a sustained...usually formalized (ie, program-based), developmental relationship” [31], in which the mentor has more experience than the mentee and the goal is the development of the mentee. As the literature often discusses interventions together, we refer to both with the umbrella term “peer-based interventions,” an appropriate appellation because of the similarities between the “active ingredients” of these 2 types of interventions. Both offer opportunities for role modeling, sharing of personal stories and practical strategies (“peer-based information”), emotional support, and encouragement from patients [30,32,33].

Prior research shows promise for peer-based interventions with complex chronic illnesses [34-36]—including ESKD—within face-to-face and telephone-based modalities. For instance, face-to-face peer mentoring in hemodialysis care has promoted completion of advance directives and organ donation documentation [37,38].

Peer-based interventions are increasingly offered in digital formats that facilitate patient-to-patient connections outside of health care contexts [39,40]. Like in-person modalities, digital peer-based interventions may enhance patient activation [41] and self-management behaviors [42,43] for patients with chronic conditions, including those with ESKD [44]. Yet, little is known about how patients—such as those with ESKD on hemodialysis—take up and engage with interventions in care contexts.

### Patient Engagement With Digital Peer-Based Interventions

Understanding recruitment, engagement, completion, and attrition in patient-facing digital interventions is a central focus of digital health research [45,46] for 2 reasons: they are difficult to achieve [47,48], and they are critical precursors of intervention effects [49-52]. Furthermore, some research has shown differences in study outcomes based on which intervention elements were used, and how much (eg, the study by Arden-Close et al [53]). Therefore, following theoretical work [52], this study investigated engagement as a multidimensional concept in which peer-mentoring session attendance, educational module completion, patient goal-setting, and information sharing with other patients and family were treated as distinct, but related, indicators of engagement. Additionally, the current study investigated a subjective part of engagement [52], that is, how patients themselves evaluated their intervention experience [54,55].

Studies of recruitment, engagement, completion, and attrition in digital interventions must also consider who is recruited into, engages with, and drops out of digital health interventions [56]. Prior digital health research has raised concerns that digital interventions may unintentionally reproduce existing inequities if socially patterned differences in these factors are not proactively addressed [56]. These concerns are salient for a hemodialysis patient-focused intervention, since these patients often have disabilities, such as low vision, manual dexterity issues, or difficulty communicating; in turn, these characteristics affect the accessibility and usability of digital interventions [57,58]. Moreover, most US hemodialysis patients are older adults [2], a group with reduced technology access and experience when compared to their younger counterparts [57]. The results from this project [59] and others [60,61] have shown that providing technology access, training, and/or technical support can facilitate the participation of medically complex patients and/or older adults. Prior work has not typically investigated the equity of digital intervention recruitment, engagement, completion, and attrition among medically complex and typically older patient populations where technology support is available, but this component is important, since support may remove some of the oft-cited barriers.

### Social Diffusion and Information Sharing

Digital health interventions are typically assessed at the individual patient level. However, patients are generally part of social networks involving family members, friends, and other patient peers—especially in in-center hemodialysis, since treatment is ongoing and usually delivered in a group setting. Prior research on distributed health literacy suggests that health knowledge and literacy-related practices are often shared across networks, rather than residing solely within individuals [62,63]. Other research links patients’ information sharing with members of their networks to their own behavior changes. Such information sharing may occur due to a patient’s desire to positively influence others’ behavior, while also reinforcing a personal commitment or motivation to engage in the behavior, partly to serve as a role model. Despite these potential social and network-mediated effects, relatively little digital health intervention research has examined how intervention content may diffuse through patients’ interpersonal networks or how participants may share intervention-related information and support with others.

To address these gaps, the Dialysafe study investigated the following research questions:

- Research question 1: Which patients participated in the intervention, and what factors were associated with recruitment, engagement, intervention completion, and attrition?
- Research question 2: How did mentees engage with the educational, goal-setting, and peer-mentoring components of the intervention, and how much did they share intervention material with other patients and family?
- Research question 3: What changes in health-related knowledge, behaviors, and sharing of intervention content did mentees report?
- Research question 4: How did mentees and peer mentors evaluate their experience of the intervention? What changes did they recommend toward improving the intervention in the future?

## Methods

### Study Design

This paper reports on a mixed methods study of a digital patient activation intervention implemented as part of a cluster-randomized, factorial controlled trial in 10 hemodialysis facilities. The study research questions are addressed using electronic health record (EHR) demographic and clinical data, intervention-platform usage data, postintervention surveys, and qualitative notes from clinic operations committee meetings. The trial is registered at ClinicalTrials.gov (NCT03171545).

### Description of the Intervention

#### Overview

Dialysafe was a cluster-randomized clinical trial comparing a digital patient activation intervention and a multimodal provider intervention. Five clinics received only the digital patient activation intervention, and 5 received both the patient activation and provider-facing interventions [64]. The digital patient activation involved education and peer mentoring [21], completed via a tablet, the Peer Mentoring Management System (PMMS). The tablet included 5 educational modules and goal-setting modules and a videoconferencing platform for speaking to a peer mentor after the completion of each module. Participation in the digital patient activation intervention was optional and did not impact collection of clinical outcomes data for the Dialysafe study.

The digital patient activation intervention was grounded in social cognitive theory and self-determination theory. In social cognitive theory, self-efficacy, expectations about the likelihood of a valued result, environmental barriers or facilitators, and goals contribute to an individual’s motivation to complete health behaviors [65]. Motivation, in turn, promotes engagement in health behavior. Peer mentors in the digital patient activation intervention aimed to impact mentees’ self-efficacy through role modeling; they also led discussions about goals set by mentees in each educational module. Self-determination theory constructs, including autonomy and relatedness [66], were targeted through autonomy support integrations in the user interface of the patient-facing intervention [21]. For instance, mentees themselves chose their own module-specific goals.

Mentors were trained in motivational interviewing techniques [67] to promote mentee health behavior. Initially, an online activity prompted mentees to identify 3-4 important values (of 19), including options such as “independent” or “responsible.” This information was intended for the mentor’s use to help motivate mentees toward reaching their goals.

#### Educational Modules

Each educational module covered a topic including the following: (1) getting enough dialysis; (2) feeling better with less salt/sodium; (3) making fluid restrictions work for you; (4) feeling better on dialysis and having easier sessions; and (5) getting more involved in your care. Each module had the same elements: slides presented as a video with narration by a member of the National Kidney Foundation (NKF), a 3-question multiple-choice quiz covering information from the slides, a goal-setting module, video clips of patients describing their experience with the topics covered in the slides (“patient stories”), and PDF handouts that could be viewed on the tablet and/or emailed to the mentee’s email address on file (Figure 1). Each goal-setting module comprised 5 questions. Mentees were asked to rank, on a scale of 1-10, the importance of the topics covered in the module and their confidence in their ability to perform them. Next, they were asked to select a goal from a list of 6-9 health behavior options, or to write in their own. Finally, they were asked when they would start their health behavior and how often they would perform it.

**Figure 1:**
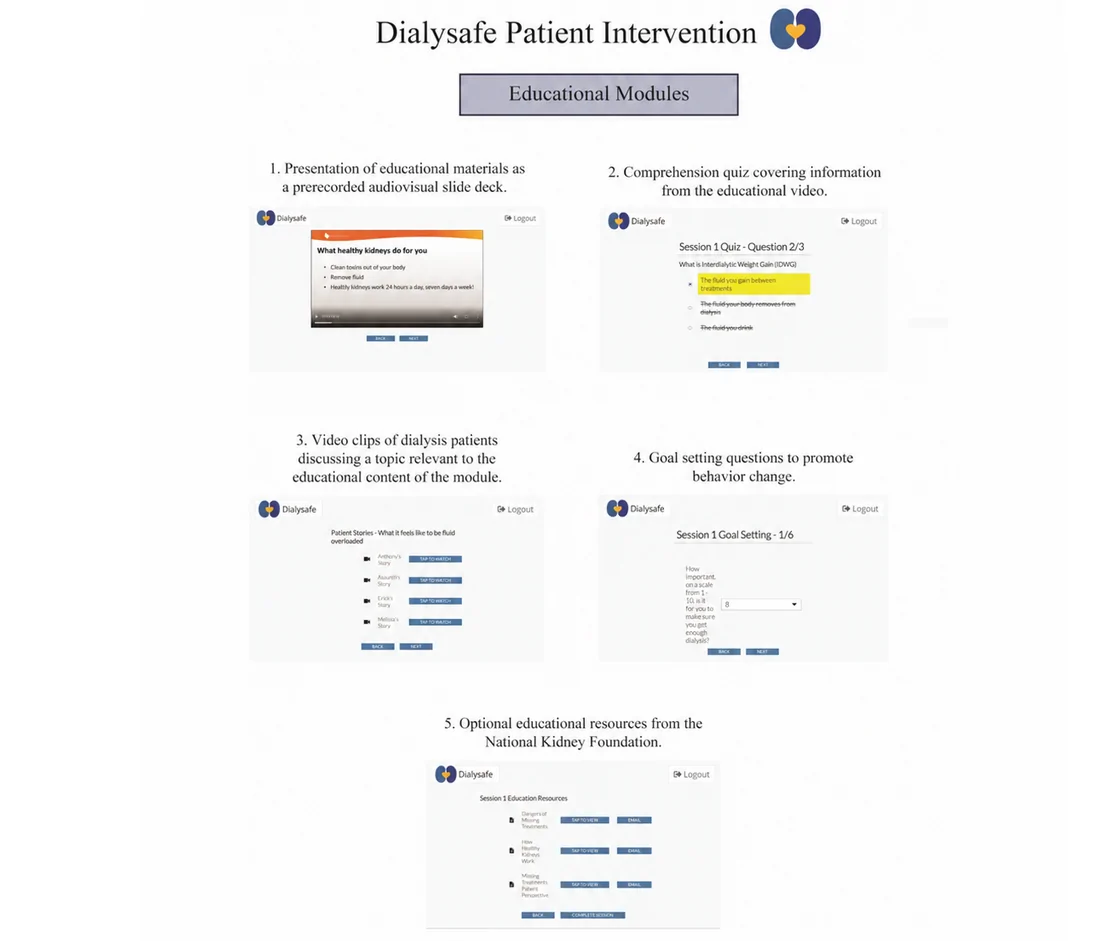
Overview of Dialysafe educational modules. IDWG: interdialytic weight gain.

#### Peer Mentoring

Peer mentors were recruited and trained by NKF. All mentors were patients with ESKD who had received in-center hemodialysis. Mentor training was adapted from NKF mentoring programs to include information specific to the Dialysafe study, including motivational interviewing techniques, as well as teaching skills and role-modeling. Mentors were introduced to the PMMS for mentoring sessions and matched to mentees based on availability and mentee preferences for their mentor’s gender.

Peer-mentoring sessions were scheduled for 5 weeks. During each peer-mentoring session, the mentor completed a discussion guide based on the mentee’s answers, with a discussion of barriers that the patients may experience while trying to reach their goals. At the end of the session, the discussion guide was submitted as an “action plan” to the mentee’s email address. Meetings were rescheduled if the mentee or mentor was unable to attend.

#### Technology Provision and Onboarding

Patients enrolled in the digital patient activation intervention (“mentees”) received a free, internet-capable tablet computer. This tablet was configured so that only the PMMS website and the necessary accessibility features were available. Mentees received a family-and-friends information sheet, charging cable, and quick-start guide. Wi-Fi hotspots were provided as needed for mentees. Technology training calls were scheduled with each mentee 1 week after shipment of the tablet to guide mentees through use of the tablet and the online PMMS system. A staffed telephone helpline was available during working hours, or as scheduled, for help with questions or difficulties encountered over the course of the intervention [59].

At the start of intervention delivery with the first 4 clinics, and despite the availability of technical support, a technical issue with videoconference platform integration delayed intervention start by a few weeks. During this period, many patients had their tablets but could not have peer-mentoring sessions—although they had access to the educational modules.

### Mentees

#### Eligibility

Eligibility for the Dialysafe study has been previously described [64]. Briefly, patients receiving in-center hemodialysis for ESKD at participating clinics were enrolled in the Dialysafe study if they did not meet the following exclusion criteria: (1) <21 years of age; (2) transient (not a permanent patient); (3) incarcerated; (4) poor cognition based on clinician judgment or failure to complete the quality of life assessment due to cognitive impairment; (5) unable to comprehend the patient information sheet in English or Spanish; or (6) patient request not to be enrolled. Patients could opt out of the overall study at any time and were unenrolled if they stopped receiving in-center hemodialysis at the participating clinic. Any enrollment in the Dialysafe study, regardless of intervention arm, meant that dialysis sessions and clinical data were included in the measurement of study outcomes, mediators, moderators, and covariates. Enrollment in the digital patient activation intervention was conducted after enrollment into the overall Dialysafe study.

#### Recruitment Into the Digital Patient Activation Intervention

All English-speaking patients enrolled in Dialysafe at clinics assigned the digital patient activation intervention were eligible to participate in the digital patient activation intervention. Within each facility, due to funding and operational constraints, the goal was that a maximum of 30 patients would receive the intervention. The recruitment approach was to select patients at random from among those who were not vulnerable and had not opted out of the study, until 30 patients had accepted the intervention. However, due to the large number of patients who could not be reached or declined, peer mentoring was offered to every eligible patient, thus undermining the random selection.

Once patients were reached, this study’s coordinator recruited enrolled patients into the intervention via phone call, during which patients gave informed consent.

To facilitate nursing home residents’ participation, this study’s team developed a letter to be sent to the Director of Nursing and the Medical Director at the nursing home for any resident who agreed to participate in the patient activation intervention. The letter stated that this study would provide a tablet and gave nursing home staff information about tablet delivery and requirements for internet access, charging, storage, and a private space for peer-mentoring sessions. It was stated that this study team would provide training and technical support and arrange return shipping, while nursing home staff could assist with tablet use if comfortable.

### Data Collection

#### EHR Data

EHR data included demographic, dialysis session, and clinical data for patients enrolled in Dialysafe. Demographic data included age, race, ethnicity, sex, and education level. Clinical outcome data included length of time on dialysis, catheter access, and comorbidities.

#### PMMS Data

PMMS data were stored on a secure server at Michigan Medicine, the University of Michigan-affiliated health system. Usage data from PMMS, including peer mentoring start and stop dates, number of peer-mentoring sessions completed, goals set in each education module, quiz answers from each education module, and page views of educational module webpages, were linked with mentee demographic data in the EHR.

#### Group Interviews at Study Clinics

To address questions and/or concerns, 3 intermittent operations committee meetings were held with each clinic with an active intervention. These meetings were attended by the clinic manager, study staff, and a patient representative. Some of these patient representatives were study participants, but that was not a requirement. Meetings focused on clinicians’ and patients’ perspectives regarding intervention implementation in the clinics and, where necessary, included troubleshooting discussions. During these meetings, study staff asked several semistructured questions about intervention implementation and recorded the responses verbatim.

#### Survey Data

Mentors completed demographic surveys as part of their PMMS profile. Satisfaction surveys completed via Qualtrics were also sent to mentees and mentors upon completion of each mentee’s final mentoring session. Surveys assessed overall satisfaction with the digital patient activation intervention, ease of using the technical components, mentor satisfaction with training, and mentee engagement with family/friends and peers. The mentee survey had 16 questions; the mentor survey had 22 questions. Honoraria were provided upon survey completion.

### Data Analysis

#### Quantitative Data

Analyses used data from the PMMS, satisfaction surveys, and EHR.

The Charlson Comorbidity Index (CCI) measures a patient's burden of chronic conditions by accounting for multiple comorbidities and age.

The CCI does not include conditions common among hemodialysis patients: hypertension, anemia, mental health disorders, and sleep disorders [68]. Therefore, we included these conditions as binary variables in our analyses alongside the CCI.

Regarding “recruited,” this refers to patients who were contacted and agreed to participate in the digital patient activation intervention.

Regarding “adopted,” this includes patients who completed at least 1 mentoring session or at least 1 educational module element.

Regarding “completed,” this refers to whether mentees met with their mentor for all 5 mentoring sessions. The amount of educational material completed by mentees is not included in this measure because of low educational module completion among most mentees, and because mentees described the mentoring as the most valuable intervention component.

Regarding the engagement score, it was created to estimate the intervention “dose” received by each mentee using weighted points for completed components. Components were weighted according to the level of active participation they required and their intended role in supporting behavior change. Mentoring received the greatest weight because it involved sustained interpersonal participation, role modeling, motivational interviewing, and individualized discussion of goals and barriers. Goal setting received the next-highest weight because mentees selected a health behavior and specified when and how often they would perform it. Videos, patient stories, and quizzes focused primarily on exposure to or recall of information rather than individualized planning or discussion. For each of the 5 module cycles, mentees could receive 5 points for attending the corresponding peer-mentoring session, 2 points for completing the goal-setting activity, and 1 point each for viewing the educational video, completing the quiz, and viewing at least 1 patient story video. Each cycle contributed up to 10 points, producing a total score ranging from 0 (no engagement) to 50 (completion of all components for all 5 modules). The weights reflected the intended intensity of engagement rather than the estimated effects of the components.

Patient characteristics were compared based on enrollment and completion status using *t* tests and chi-square tests. Comparisons involving categorical variables with very small or 0 cell counts were reported for completeness but were not used for inferential interpretation.

Logistic regression models were fit to assess predictors of joining the intervention and completing 5 sessions as binary outcomes, respectively. Due to an overabundance of zeroes in the data, a zero-inflated Poisson model was also used to model predictors of the engagement score as a count-based outcome. Variables for these models were selected theoretically to reflect demographic and clinical factors shown to affect uptake and engagement with digital health interventions in prior research (eg, other studies [56,69]).

#### Qualitative Data

Qualitative data included notes from the operations committee meetings and free-text responses from the satisfaction surveys. These data were analyzed using NVivo (version 14; Lumivero). Inductive codes were created via an iterative process with 3 rounds of review among 3 study staff members (MW, KE, and TCV). Once a codebook was created, MW and KE both coded a randomly selected 20% of the data. Concordance was evaluated using the interrater reliability query in NVivo; a Cohen κ of 0.7 indicated substantial agreement. MW and KE then coded the remaining data. Because the qualitative sample comprised all available operations committee notes and free-text survey responses, saturation was not used as a data-collection stopping criterion. All available qualitative data were analyzed through iterative coding, and only themes with sufficient support in the dataset were reported. Qualitative notes were used to answer study research questions as part of a mixed methods approach.

#### Mixed Methods Analysis

Per a convergent mixed methods approach [70], data were analyzed separately and then mixed in later analyses. MW created joint displays [71] that presented qualitative and quantitative data.

### Ethical Considerations

This study was approved by the institutional review board (HUM00125305). Although a waiver of consent and HIPAA (Health Insurance Portability and Accountability Act) authorization was granted to enroll participants in Dialysafe data collection, patients were provided an informational sheet and given a minimum of 1 week to decline before being enrolled. Recruitment for the digital patient activation intervention used the pool of enrolled patients at clinics assigned that intervention. During recruitment, patients provided verbal consent to join the digital patient activation intervention. Patients could drop out of the digital patient activation intervention or data collection from the Dialysafe study at any time. Data collected before withdrawal remained in the analyses unless participants requested their removal. Both mentors and mentees were compensated for their participation. Peer mentors received US $100 for each mentee who completed the program, paid after the program was completed. Mentees received payments totaling US $120 (US $10, US $15, US $20, US $25, and US $50 for sessions 1-5, respectively, with the payment contingent on completing the satisfaction survey) and were sent a blanket after their first meeting with a mentor.

Data were stored on secure servers. Patients were assigned a randomly generated study identification number to link records across data files.

## Results

### Research Question 1: Which Patients Participated in the Digital Patient Activation Intervention, and What Factors Were Associated With Recruitment, Engagement, Intervention Completion, and Attrition?

#### Recruitment Into the Intervention

Although all eligible patients received up to 10 (mean 2.9, SD 2.3) recruitment phone calls, a subset of patients were recruited; the remaining patients declined to join or were not reached despite multiple call attempts. Of the 639 eligible patients across 10 clinics, only 155 (24.3%) were recruited into the intervention, while 272 (42.6%) declined to join. A third of the 272 patients who declined to join the intervention did so because they were not interested in peer mentoring (90/272, 33%; see Multimedia Appendix 1 for details). The remaining 212 patients of the 639 (33.2%) participants who were eligible could not be contacted after multiple attempts. Reflecting on recruiting via phone call, the clinic manager for clinic J said: “Most people don’t really chitchat on the phone as often as they used to. Especially with strangers. Even when they don’t show up for their treatments, we have to call like 4 different emergency contacts to get to them.”

Of the 155 (24.3%) mentees recruited to the digital patient activation intervention, the average age was 57.6 (SD 13.8) years, and 87 (56.1%) mentees were male (Table 1). There were no significant racial or ethnic differences between participants and nonparticipants; most participants in both groups were White or Black/African American. Among patients not recruited to the digital patient activation intervention, the average age was 64.3 (SD 14.7) years (*P*<.001), and 283 (58.5%) patients were male. Mentees recruited to the digital patient activation intervention had slightly lower CCI scores compared to those who did not join the intervention but were no more likely to be within their first year of dialysis treatment. Most eligible patients had at least a high school education; those who were recruited to the digital patient activation intervention were more likely to have attended some college or technical school (*P*=.03). We also note that despite the existence of procedures for nursing home residents to participate in the patient activation intervention, no one identified as currently living in a nursing home was recruited.

A logistic regression was performed to determine patient characteristics that were associated with recruitment into the intervention (Table 2). As shown, each year of age was associated with 4% lower odds of recruitment (odds ratio [OR] 0.96, 95% CI 0.94-0.98; *P*<.001). Patients with a college degree were more likely to be recruited into the digital patient activation intervention (OR 1.70, 95% CI 1.02-2.83; *P*=.04). Although comorbidities had no impact on the likelihood of recruitment, patients with diagnosed sleep disorders were more likely to be recruited (OR 2.27, 95% CI 1.06-4.87; *P*=.03). As the intervention was offered to all eligible patients, these associations reflect the combined likelihood of being reached and agreeing to participate, rather than selection under the intended random-selection procedure.

**Table 1:** Demographics of mentee recruitment.

			Patients recruited into the digital patient activation intervention (n=155)		Patients who were not recruited into the patient activation intervention (n=484)		*P* value^a^
**Age (years), mean (SD)**							
	Age at time of enrollment	57.6 (13.8)		64.3 (14.7)		<.001^b^	
**Sex, n (%)**							
	Male	87 (56.1)		283 (58.5)		.61	
	Female	68 (43.9)		201 (41.5)			
**Race, n (%)**							
	Black/African American	60 (38.7)		161 (33.3)		.22	
	White	85 (54.8)		295 (61.0)		.18	
	Asian	1 (0.6)		8 (1.7)		.25	
	Native Hawaiian/other Pacific Islander	1 (0.6)		1 (0.2)		.52	
	Other	3 (1.9)		8 (1.7)		.81	
	Unknown/missing	5 (3.2)		11 (2.3)		.55	
**Ethnicity, n (%)**							
	Hispanic	19 (12.3)		81 (16.7)		.18	
**Highest education achieved, n (%)**							
	Less than high school	11 (7.1)		75 (15.5)		.002^b^	
	High school graduate or GED^c^	55 (35.5)		197 (40.7)		.25	
	Some college or technical school	49 (31.6)		110 (22.7)		.03^b^	
	College graduate	40 (25.8)		102 (21.1)		.22	
**Clinical characteristics**							
	Charlson Comorbidity Index, mean (SD)	3.2 (2.2)		3.8 (2.3)		.004^b^	
	Sleep disorder, n (%)	14 (9.1)^d^		20 (4.2)^e^		.05	
	Mental health disorder, n (%)	27 (17.5)		71 (15.1)		.47	
	Anemia, n (%)	124 (80.5)		360 (76.4)		.28	
	Hypertension, n (%)	119 (77.3)		345 (73.2)		.32	
	Dialysis vintage <1 year, n (%)	32 (20.6)		81 (16.7)		.29	
	Catheter access, n (%)	28 (18.1)		109 (22.5)		.24	
	Missing vascular access, n (%)	5 (3.2)		12 (2.5)		.64	
	Diabetes as cause of ESKD^f^, n (%)	65 (41.9)		203 (41.9)		.99	
	Missing cause of ESKD, n (%)	2 (1.3)		14 (2.9)		.18	

^a^*P* values were calculated using *t* tests for continuous variables and chi-square tests or Fisher’s exact tests, as appropriate, for categorical variables.

^b^*P*<.05

^c^GED: General Educational Development.

^d^One person in this group had missing comorbidity data, so the denominator for comorbidities in this column is n=154.

^e^Thirteen people in this group had missing comorbidity data, so the denominator for comorbidities in this column is n=471.

^f^ESKD: end-stage kidney disease.

**Table 2:** Predictors of recruitment into, engagement with, and completion of the digital patient activation intervention.

Demographics		Recruitment into patient activation intervention (n=639; 155 recruited; logistic regression)		Engagement with intervention (n=155; zero-inflated Poisson model)			Completed intervention (ie, finished 5 mentoring sessions; n=155; 58 completed; logistic regression)	
		Odds ratio (95% CI)	*P* value	Incidence rate ratio (95% CI)	*P* value	Odds ratio (95% CI)		*P* value
						
**Age (years)**								
	Age at time of enrollment (per year)	0.96 (0.94-0.98)	<.001^a^	1.00 (0.998-1.01)	.50	0.95 (0.91-0.99)		.01^a^
**Sex (reference: female)**								
	Male	0.87 (0.59-1.29)	.50	1.33 (1.22-1.45)	<.001^a^	2.03 (0.93-4.43)		.08
**Race (reference: White)**								
	Black/African American	1.02 (0.66-1.58)	.91	1.27 (1.16-1.39)	<.001^a^	0.65 (0.28-1.51)		.31
	Asian	0.33^b^ (0.04-2.90)	.31	0.02^b^	.87	<0.001^b^		>.99
	Native Hawaiian/other Pacific Islander	3.60^b^ (0.20-65.92)	.39	0.09 ^a^ (0.01-0.64)	.02^a^	<0.001^b^		>.99
	Other race	1.62 (0.36-7.34)	.53	1.14 (0.76-1.70)	.52	6.01^b^ (0.22-165.58)		.29
	Missing race	0.93 (0.27-3.22)	.91	0.68 (0.53-0.87)	.002^a^	0.57 (0.06-5.36)		.63
**Ethnicity (reference: non-Hispanic)**								
	Hispanic	0.68 (0.35-1.32)	.26	0.97 (0.82-1.15)	.71	0.47 (0.11-1.96)		.30
**Highest education achieved (reference: high school graduate/GED** ^c^ **)**								
	Less than high school	0.66 (0.31-1.38)	.27	1.09 (0.92-1.30)	.33	1.09 (0.20-6.07)		.92
	Some college education	1.39 (0.86-2.25)	.18	1.17 (1.05-1.29)	.003^a^	1.40 (0.54-3.62)		.48
	College graduate	1.70 (1.02-2.83)	.04^a^	1.02 (0.90-1.15)	.77	0.97 (0.36-2.61)		.95
**Clinical characteristics**								
	Charlson Comorbidity Index (per 1 unit)	1.01 (0.89-1.15)	.86	1.04 (1.01-1.07)	.002^a^	1.37 (1.05-1.79)		.02^a^
	Sleep disorder^d^ (yes vs no)	2.27 (1.06-4.87)	.03^a^	0.97 (0.82-1.15)	.72	0.62 (0.15-2.68)		.52
	Mental health disorder^d^ (yes vs no)	1.06 (0.62-1.82)	.83	1.10 (0.98-1.23)	.12	0.35 (0.10-1.15)		.08
	Anemia^d^ (yes vs no)	1.17 (0.71-1.92)	.55	2.06 (1.79-2.37)	<.001^a^	0.90 (0.31-2.58)		.84
	Hypertension^d^ (yes vs no)	1.07 (0.67-1.72)	.76	0.97 (0.87-1.09)	.63	1.40 (0.52-3.82)		.51
	Dialysis vintage <1 year (reference: ≥1 year)	1.54 (0.85-2.79)	.16	1.22 (1.05-1.42)	.01^a^	1.53 (0.44-5.31)		.50
	Catheter access (reference: no catheter access)	0.59 (0.34-1.03)	.06	0.83 (0.72-0.97)	.02^a^	0.41 (0.12-1.45)		.17
	Missing vascular access	0.77 (0.22-2.66)	.68	0.92 (0.75-1.13)	.42	0.30 (0.02-3.85)		.35
	Diabetes as cause of ESKD^e^	1.24 (0.82-1.89)	.31	0.95 (0.87-1.05)	.31	0.73 (0.31-1.71)		.46
	Missing cause of ESKD	0.44 (0.09-2.13)	.30	0.005^b^	.79	>999^b^		.99

^a^*P*<.05.

^b^These estimates are unstable due to small sample sizes.

^c^GED: General Educational Development.

^d^For binary clinical characteristics, the reference category was the absence of the condition.

^e^ESKD: end-stage kidney disease.

#### Engagement

The weighted engagement score was calculated for all mentees recruited to the intervention (Table 3). Scores ranged from 0 to 50. However, the mean value was approximately 3 times the median (mean 18.37, SD 20.05, median 6.0, IQR 0-41). The distribution was highly skewed, with a large mass at 0, reflecting the high number of recruited participants who never engaged with any intervention components. This is notable, since most recruits (139/155, 89.7%) received the study-specific tablets with the educational modules and videoconferencing platform preloaded on them. Some of this preintervention attrition may be linked to a technical issue that delayed intervention start by a few weeks in the first 4 study clinics.

**Table 3:** Overall engagement score for patients recruited to the patient activation intervention (N=155). Engagement scores were weighted composite scores ranging from 0 to 50, with higher scores indicating greater engagement with intervention components: mean 18.37, SD 20.05, median 6.0, IQR 0-41; range 0-50. Score ranges are left-open-right-closed intervals except the first, which includes 0. Percentages may not sum to 100 due to rounding.

Engagement score range	Recruited mentees, n	Recruited mentees (%)	Cumulative (%)
0-5	77	49.7	49.7
5-10	4	2.6	52.3
10-15	4	2.6	54.8
15-20	6	3.9	58.7
20-25	2	1.3	60.0
25-30	2	1.3	61.3
30-35	6	3.9	65.2
35-40	13	8.4	73.5
40-45	24	15.5	89.0
45-50	17	11.0	100.0
Total	155	100.0	N/A^a^

^a^N/A: not applicable.

Although several mentees did not engage with any components, those who did engage were likely to complete the majority of components (41/78, 53%, engagers scored above 40; Table 3). However, the respondent for mentor survey #41 reported, “Most of the time, the mentee has not read any materials.”

Among those recruited to the intervention, males had higher overall engagement scores than females (incidence rate ratio [IRR] 1.33, 95% CI 1.22-1.45; *P*<.001), as did Black/African American compared with White individuals (IRR 1.27, 95% CI 1.16-1.39; *P*<.001), mentees receiving dialysis for less than one year compared to those on dialysis for more than a year (IRR 1.22, 95% CI 1.05-1.42; *P*=.01), and mentees with a diagnosis of anemia compared to those without (IRR 2.06, 95% CI 1.79-2.37; *P*<.001). Mentees with some college or technical school education were the only education group to have higher overall engagement scores than mentees with a high school education (IRR 1.17, 95% CI 1.05-1.29; *P*=.003; Table 2).

#### Completion

A total of 58/155 completed the intervention (ie, all 5 mentoring sessions), which amounted to 58 of 639 (9.1%) eligible patients. Mentees with more comorbidities per the Charlson index were more likely to complete all 5 sessions (OR 1.37, 95% CI 1.05-1.79; *P*=.02) and to have higher engagement scores (IRR 1.04, 95% CI 1.01-1.07; *P*=.002). In contrast, lower overall engagement scores were likely for those using a catheter for access (IRR 0.83, 95% CI 0.72-0.97; *P*=.02) or whose race was not listed (IRR 0.68, 95% CI 0.53-0.87; *P*=.002; Table 2). Engagement scores were lower among Native Hawaiian/other Pacific Islander mentees (IRR 0.09, 95% CI 0.01-0.64; *P*=.02); although this association was significant, the number of Native Hawaiian/other Pacific Islander participants was very small (n=1), and the corresponding result should therefore be interpreted cautiously.

Logistic regression showed that younger mentees were more likely to finish all 5 mentoring sessions (OR 0.95, 95% CI 0.91-0.99; *P*=.01). Each additional year of age was associated with lower odds of completing all 5 mentoring sessions (OR 0.95, 95% CI 0.91-0.99; P=.01).

#### Attrition

#### Clinic Patients

Of the 155 mentees recruited to the digital patient activation intervention, 93 mentees completed the technology training call; 4 mentees opted out of training. Of the mentees who completed that training, 77 mentees scheduled peer-mentoring sessions, and 72 mentees completed one or more of these sessions. See Multimedia Appendix 2 for attrition reasons.

Just under half of the mentees (72/155, 46.5%) completed 1 or more peer-mentoring sessions, a component of the patient activation intervention. In general, mentees who had met with their mentor were unlikely to discontinue the intervention; 14 of 155 (9%) mentees completed between 1 and 4 mentoring sessions.

Multimedia Appendix 3 displays demographic data for mentees by their intervention completion and attrition status. The proportion of men was higher among completers than noncompleters (40/58, 69% vs 47/97, 48.5%; *P*=.01); hence, attrition was higher among female mentees. As several racial categories contained very few observations, the corresponding comparisons were not interpreted.

#### Patient Peer Mentors

Mentors were predominantly female, Black/African American or White, and not Hispanic (Multimedia Appendix 4). Mentors who led at least 1 mentoring session were demographically similar to the overall pool of recruited and trained mentors (Multimedia Appendix 4). Among mentors who mentored at least 1 mentee, the average age was 52.4 (SD 12.8) years. Among the 11 mentors who were unable to work with mentees, scheduling conflicts were the primary barrier.

### Research Question 2: How Did Mentees Engage With the Educational, Goal Setting, and Peer Mentoring Components of the Digital Patient Activation Intervention, and How Much Did They Share Intervention Material With Other Patients and Family?

#### Interactive Educational Modules

Demographics based on engagement with the educational modules are displayed in Multimedia Appendices 5 and 6. As educational modules and peer mentoring were completed separately, engagement with the educational components does not necessarily align with peer-mentoring completion (Table 3). Among all recruited mentees (n=155), 64 (41.3%) mentees completed at least 1 quiz, 79 (50.9%) mentees completed at least 1 goal-setting module, and 82 (52.9%) mentees watched at least 1 educational video (Multimedia Appendix 6). Completion rates adjusted for the number of mentoring sessions attended are shown in Multimedia Appendix 7.

#### Education and Patient Story Videos

Each educational module began with an approximately 15-minute, prerecorded video presentation on the module’s topic. A total of 82 of 155 (52.9%) mentees watched at least 1 such video (Multimedia Appendix 6).

Each educational module also included 2-5 “patient story” videos. These were shorter video clips (approximately 2 minutes) taken from interviews with patient partners who described their experiences with each of the module topics. Only 43 of 155 (27.7%) mentees watched at least 1 patient story video (Multimedia Appendix 6).

Overall, mentees rated each of the module topics above 9, indicating that they believed the topics were very important (Multimedia Appendix 8). Mentees rated the sessions “feeling better on dialysis and having easier sessions” and “getting more involved in your care” as most important (mean of 9.5, SD 1.6, and 9.9, SD 0.5, respectively). Participants were slightly less confident in their abilities to perform behaviors related to the module theme; the lowest score was for “making fluid restrictions work for you” (mean of 8.5, SD 2.4; Multimedia Appendix 8).

#### Quizzes

A total of 64 of 155 (41.3%) mentees completed at least 1 quiz from the educational modules, with most completing all 5 quizzes. Although some mentees completed quizzes for which they did not later attend a corresponding mentoring session, most quizzes (209 quizzes / 319 mentoring sessions × 100 = 65.5%) were completed by mentees who also attended the mentoring session.

#### Goal-Setting Modules

The first goal-setting section also included a “values identification” question to assist with motivation (Multimedia Appendix 9). A total of 76 mentees selected their top 3-4 values from a list of 19 provided. The top 3 values indicated wanting to be independent (54/76, 71.1% of mentees), strong (29/76, 38.2% of mentees), and responsible (25/76, 32.9% of mentees). A mentee’s identified values were revisited at each mentoring session to motivate the “why” underlying their goal setting and behavior change.

Each goal-setting module then asked mentees to rate, on a scale from 1-10, the importance of the topic covered in the module and their confidence in their ability to perform health behaviors related to the topic. They then selected a goal from a list of multiple-choice options (or wrote in their own) covering health behaviors related to the module’s topic. Finally, mentees identified how often they would perform their selected goal and when they would start. A total of 79 of 155 (51%) mentees answered all goal-setting questions from one or more educational modules.

Mentees set goals in four main categories: (1) communication with care team, (2) staying for all scheduled sessions or feeling more comfortable at dialysis, (3) limiting sodium and/or fluid, and (4) tracking health metrics and behaviors (Table 4). Detailed health behavior goals selected by mentees during the goal-setting component of each educational module are also shown in Multimedia Appendix 10.

**Table 4:** Mentee goals and self-reported behavior changes relevant to dialysis session stability—joint display of qualitative and quantitative data.

Patient involvement in intradialytic hypotension prevention			Supportive qualitative data	Supportive quantitative data, n/N (%)
**Communication with care team**				
	Asking questions, sharing concerns about dialysis treatment with care team	Additional conversations between patients and staff: “I’ve noticed that on occasion there has been more discussion for patients who are more engaged and interested in their care. They’re able to articulate their questions in a more meaningful way that we can answer.” (clinic manager, clinic N) “The patients know more...they have more knowledge about hypotension. They’re kind of able to judge their fluid intakes and the nurses are able to know the patients’ full symptoms when they’re not in the clinic.” “[A patient who recently started the peer mentoring program] was concerned about weight gains and shortness of breath and things like that.” (clinic manager, clinic T) Unsure about additional conversations “I haven’t heard much from the patients.” (clinic manager, clinic F) Interviewer: “Have there been any changes in topics that the patients bring up?” Clinic manager: “I wouldn’t be the one to hear that. I’m not the one who does the assessment; my senior nurse would be the one they like to go to.” (clinic P)		Goal setting during intervention 64/79 (81%) patients set a goal to talk to their care team about symptoms, questions, and/or concerns regarding their dialysis treatment (multiple responses possible). Reported behavior change 10/54 (18.5%) patients identified a behavior change to keep their care team informed about changes in their health, such as emergency department trips, upcoming procedures, or medication changes. 6/54 (11.1%) patients identified a behavior change of reporting symptoms during their dialysis session to dialysis staff. 4/54 (7.4%) patients reported talking to their dialysis team about their fluid removal goal or ultrafiltration rate. 2/54 (3.7%) patients reported talking to their dialysis team about their estimated dry weight.
**Staying for all scheduled sessions or feeling more comfortable on dialysis**				
	Fewer missed/shortened sessions	N/A		Goal setting during intervention 48/79 (61%) patients set a goal to reduce their amount of missed dialysis time and/or sessions (multiple responses possible). Reported behavior change 4/54 (7.4%) patients indicated on their surveys that the main behavior change that they made as a result of the intervention was not shortening or skipping dialysis sessions.
	Finding a distraction from dialysis treatment, putting feet up during treatment, or bringing comfort items (eg pillow or blanket)	N/A		Goal setting during intervention 16/79 (20%) patients set a goal to feel more comfortable at their dialysis treatment (multiple responses possible).
**Limiting sodium and/or fluid**				
	Limiting sodium intake	Mentee survey: What did you like best about the Dialysafe Program? Respondent #27: “Discussing tips or tricks on how to limit/reduce fluid intake, sharing different renal diet recipes or tips on creating better kidney friendly foods.”		Goal setting during intervention 41/79 (52%) patients set a goal to reduce their sodium intake (multiple responses possible). Reported behavior change 8/54 (14.8%) patients indicated on their surveys that the main behavior change that they made as a result of the intervention was to limit their sodium intake.
	Limiting fluid intake	Interviewer: “Have you noticed any changes to your health since the study began?” Patient representative: “I just went to Florida and went three days without dialysis. That’s a lot for me. But I watched my fluid so I wasn’t as high on Monday as I was expecting.” (clinic J)		Goal setting during intervention 32/79 (41%) patients set a goal to reduce their fluid intake (multiple responses possible). Reported behavior change 5/54 (9.3%) patients indicated on their surveys that the main behavior change that they made as a result of the intervention was to limit their fluid intake.
**Tracking health metrics and behaviors**				
	Tracking home blood pressures	Bringing home blood pressure readings to clinic “[We have] several people doing blood pressures at home for the first time.” (clinic manager, clinic D) Not bringing home blood pressure readings to clinic Interviewer: “Have patients brought in any home measurements – blood pressures, weights?” Clinic manager: “Not typically. Only if they’re asked.” (clinic J) Clinic manager: “Well, they might have brought it in and spoke to the nurses about it, but I haven’t heard anything.” (clinic L)		Goal setting during intervention 19/79 (24%) patients set a goal to track their blood pressure at home (multiple responses possible). Reported behavior change 6/54 (11%) patients indicated on their surveys that the main behavior change that they made as a result of the intervention was tracking their blood pressure at home.
	Tracking weight at home	Bringing home weight readings to clinic Interviewer: “Has anyone brought in home blood pressures or weights?” Clinic manager: “We do have a few patients who do that. I think some of them weren’t ones who were doing it before.” (clinic P) Not bringing home weight readings to clinic “No patients bring up...blood pressures at home, weights at home.” (clinic manager, clinic B)		Goal setting during intervention 11/79 (14%) patients set a goal to track their weight at home (multiple responses possible). Reported behavior change 2/54 (3.7%) patients indicated on their surveys that the main behavior change that they made as a result of the intervention was tracking their weight at home.
	Tracking sodium intake	N/A		Goal setting during intervention 10/79 (13%) patients set a goal to track sodium intake and/or read ingredient labels while shopping (multiple responses possible). Reported behavior change 1/54 (1.9%) patient indicated on their survey that the main behavior change that they made as a result of the intervention was tracking their sodium intake.
	Tracking fluid intake	N/A		Goal setting during intervention 29/79 (37%) patients set a goal to track the amount of fluid they drink or become familiar with the amount of water in foods they eat (multiple responses possible). Reported behavior change 6/54 (11%) patients indicated on their surveys that the main behavior change that they made as a result of the intervention was tracking fluid intake.

Regarding communication with the care team, overall, 81% (64/79) of mentees set a goal in one or more education modules related to “communication with care team,” the highest percentage out of any category. Goals in this category included telling a member of their dialysis team about a symptom that they are experiencing (34/65, 52.3%; Multimedia Appendix 10) or sharing a health concern with a team member (22/59, 37.3%; Multimedia Appendix 10). Although goals in this category were available for selection in each educational module, module 5 (“Getting More Involved With Your Care”) contained the most directly applicable lessons and information.

Regarding staying for all scheduled sessions or feeling more comfortable at dialysis, most mentees (61/79, 77.2%) set one or more goals related to this topic (Multimedia Appendix 10). This category’s main goal was “stay for all of my scheduled sessions” (40/77, 52%, 40/79, 50.6%).

Regarding limiting sodium and/or fluid*,* across all modules, 51.8% (41/79) of mentees set a goal to limit sodium intake, while 40.5% (32/79) set a goal to limit fluid intake. The most common specific goals among the 71 participants who completed the sodium module were “replace salt with another spice” (14, 19.7%). For the 66 participants who completed the fluid restrictions module, the most common specific fluid limitation goal was “try a new method for controlling my thirst” (eg, lemon drops, frozen grapes, and ice chips; 15, 22.7%).

Regarding tracking health metrics and behaviors, this category included goals about tracking fluid intake or the amount of water in food (29/79, 36.7%, mentees), tracking home blood pressures (19/79, 24.1%, mentees), tracking weight at home (11/79, 13.9%, mentees), and tracking sodium intake (10/79, 12.7%, mentees).

#### Peer Mentoring

While mentoring sessions were initially scheduled once per week for 5 weeks, mentees and/or mentors often needed to reschedule one or more sessions. Common reasons for rescheduling included mentee no-show, medical appointment or hospitalization, travel, and technical difficulties (eg, power/internet outage or difficulty joining video call). Therefore, it took an average of 34.9 (SD 12.1) days to complete all mentoring sessions.

In total, 15 mentors mentored 72 mentees. On average, each mentor was paired with 4.80 (SD 2.45) mentees, provided 21.3 (SD 11.4) mentoring sessions, and sent 16.8 (SD 12.8) action plans to their mentees.

#### Mentee Information Sharing With Peers, Family, and Friends

More than half (32/55, 58.2%) of mentees who completed the satisfaction survey reported telling other patients in their clinic about what they learned in the digital patient activation intervention, but only 20.4% (11/54) of those survey respondents said they had other mentees talk to them about what they had learned (Table 5). Qualitative data from patient representatives in the operations committee meetings ranged from sharing intervention content with multiple other patients to sharing with just one other person whom they knew well (Table 5). One mentee who participated in the operations committee meetings also overheard others speaking about their participation. With respect to not speaking with others about what they had learned, 3 mentees referenced difficulties due to being on different shifts or not seated close to one another (Table 5).

**Table 5:** Sharing information with peers—joint display of qualitative and quantitative data.

Survey responses		Supportive qualitative data	Supportive quantitative data, n/N (%)
**Did you tell other patients in your clinic about things you learned from the educational or peer mentoring sessions?**			
	Yes	Patient representative: “I’ve talked about it with other patients but haven’t met anyone else doing the program.” (clinic H) Patient representative: “I usually talk a lot with [Name]...[Name] did ask about the study. I told him a lot about what we could drink.” (clinic J) Patient representative: “And some patients were talking about it and I thought they learned extremely well.” (clinic N)	32/55 (58%) mentees who completed the satisfaction survey said they discussed what they learned in the patient activation intervention with their peers. 11/54 (20%) mentees who completed the satisfaction survey said other patients in their clinic shared things they learned from the patient activation intervention. Six of these 11 mentees responded to the question asking, “How many people told you things?” These 6 mentees reported an average of 2.5 (SD 1.8) other people telling them things they had learned.
	No	Clinic manager: “No, not too much talking about the study. The only shift was maybe with [PATIENT REP].” (clinic J) Patient representative: “I haven’t heard from other patients. I pretty much just stay in my bubble.” (clinic P) Patient representative: “I haven’t had other patients ask me anything about it.” (clinic G) Patient representative: “Blocks in the unit are sort of like ‘neighborhoods’ and we rarely see people on the other side. We used to meet in the waiting room but now we go right to the chairs.” (clinic B)	23/55 (42%) mentees who completed the satisfaction survey said they did not discuss what they learned in the patient activation intervention with their peers. 43/54 (80%) mentees who completed the satisfaction survey said they did not hear other patients talk about what they learned from the patient activation intervention.

The majority (38/55, 69.1%) of mentees who completed the satisfaction survey indicated that they shared the “family information sheet” with one or more members of their family and/or friends (Table 6). Among the 38 mentees who shared the information sheet, the most common recipients were adult children (19/38, 50.0%), followed by friends (14/38, 36.8%), and spouses or partners (12/38, 31.6%). On average, participants agreed that their family was willing to help them reach their goals (mean 4.0, SD 1.5; Table 6).

**Table 6:** Family and friends engagement with peer-mentoring component of the patient activation intervention.

Question from satisfaction survey		Mentees, n/N (%)	Mean (SD)
**Did you share the paper titled *Patient Peer Mentoring Program: Information for Family Members of Participants* with anyone? (n=55)**			
	Yes	38 (69.1)	—^a^
	No	17 (30.9)	—
If yes, how many people did you share it with?		—	2.7 (1.6)
**If yes, whom did you share it with? (n=38)**			
	Adult child or children	19 (50.0)	—
	Friends	14 (36.8)	—
	Spouse or partner	12 (31.6)	—
	Siblings	9 (23.7)	—
	Parents	8 (21.1)	—
	Cousins	5 (13.2)	—
	Other	4 (10.5)	—
	Nieces or nephews	1 (2.6)	—
**My family was willing to help me reach my goals (1=strongly disagree; 5=strongly agree); n=55**			
		—	4.0 (1.5)

^a^Not available; multiple responses possible.

### Research Question 3: What Changes in Health-Related Knowledge, Behaviors, and Sharing of Intervention Content Did Mentees Report Following Participation in the Digital Patient Activation Intervention?

#### Patient Knowledge

While there were no preintervention knowledge quizzes, most mentees correctly answered the quiz questions after each educational module (Multimedia Appendix 11). Quiz questions ranged from 59.3% (35/59) correct (module 1, question 3) to 100% (51/51) correct (module 2, question 3); the average across all questions was 89% (SD 10.7; across 15 question-level percentages) correct. All questions except for module 1, question 3 (“Why might you need an extra, or longer dialysis session?”; correct answer=“all of the above: if you have too much fluid at the end of a dialysis session, if you missed a dialysis session, if you have been cramping during your dialysis session”) averaged higher than 77% correct (Multimedia Appendix 11 for comprehension quiz scores).

#### Health Behaviors

In the satisfaction survey, mentees were asked to identify the 1 main health behavior change that they had made, if any. Table 4 summarizes qualitative and quantitative data based on behavior changes and/or goals reported by mentees. We now discuss the major categories of behavior changes, aligned with the major categories of mentee goals described in the Goal-Setting Modules subsection.

Regarding communication with the care team, in the postmentoring survey, 40.7% (22/54) of respondents identified this category as their main behavior change after completing the digital patient activation intervention. In the operations committee meetings, some clinic managers noted changes in the clarity of questions asked by patients in the clinic and by patients sharing their symptoms between dialysis sessions (clinics N and T, Table 4). However, some clinic staff had not heard about or noticed changes (clinics F and P, Table 4).

Regarding staying for all scheduled sessions or feeling more comfortable at dialysis*,* only 7.4% (4/54) of satisfaction survey respondents indicated staying for all scheduled sessions as their main change. Dialysis adherence was discussed neither in the operations committee nor in qualitative survey responses.

Regarding limiting sodium and/or fluid*,* in total, 24.1% (13/54) of health behavior changes from the satisfaction survey pertained to sodium and fluid limits. Similarly, 2 patient representatives talked about improved ability to manage fluids in operations committee meetings (clinics J and N), and 1 survey respondent most enjoyed sharing “tips and tricks” about limiting fluid intake with their mentor (Table 4). The clinic manager from clinic B also reported that some patients asked about their fluid status in response to the weekly chairside question about recovery time.

Regarding tracking health metrics and behaviors, overall, 27.7% (15/54) of satisfaction survey answers regarding behavior change indicated tracking. Similarly, managers of 2 clinics (D and P) mentioned patients bringing metrics (eg, home blood pressure readings or weights) into the clinic, although staff in other clinics did not report this occurring (B, J, and L; Table 4).

### Research Question 4: How Did Mentees and Peer Mentors Evaluate Their Experience of the Digital Patient Activation Intervention? What Changes Did They Recommend to Improve the Digital Patient Activation Intervention in the Future?

#### Satisfaction

#### Survey Completion

Of the 58 mentees who completed all 5 mentoring sessions, 56 (96.6%) completed the satisfaction survey. Mentees who completed 1-4 mentoring sessions (n=14) were also invited to complete the satisfaction survey, but none replied. Therefore, the survey completion rate for mentees who participated in at least 1 mentoring session was 56 of 72 (77.8%). All mentors (n=15) completed at least 1 survey; 66 of 72 (91.7%) surveys from unique mentor-mentee pairings were completed.

#### Mentee-Mentor Relationship

Mentees (n=55, 1 missing response) and mentors (n=15) were highly satisfied with the intervention’s peer-mentoring component (Table 7). Mentees largely felt that their mentors were helpful, supportive, and knowledgeable, and that it was helpful to discuss their goals with the mentor (Table 7). Mentors felt that their mentees made positive changes to their health and that they were able to help their mentees (Table 7).

Overwhelmingly, mentees responded to the question “what did you like best about the Dialysafe program?” by describing mentor interactions. In fact, in 5 surveys, the answer to this question was only the mentor’s name or “my mentor.” Even more surveys provided additional information about why the respondent liked their mentor. Some mentees simply enjoyed speaking to someone new: for mentee survey #52, the respondent said the best part of Dialysafe was “meeting a new friend.” Mentees frequently cited the relationship with their mentor and the knowledge shared by their mentor as their favorite parts of the intervention (Table 7).

Overall, both mentees and mentors indicated that they would recommend the digital patient activation intervention to a friend or someone in a similar situation (Table 7). After completing the final peer-mentoring sessions, mentees could transition to the existing NKF PEERS mentoring program if they wanted continued mentoring. They were also permitted to begin the process of becoming a mentor themselves for later interventions. As clinic R’s patient representative reported, “I am thinking about starting a support group because of my time in the peer mentor program.”

**Table 7:** Mentee and mentor satisfaction with program—joint display of qualitative and quantitative data.

Survey responses			Supportive qualitative data		Supportive quantitative data, n/N (%)
**Overall, how would you rate your experience with the Dialysafe peer mentoring program?**					
	Mentees	Mentee survey: What did you like best about the Dialysafe program? Respondent #1: “Peer mentor contact, testimonials from experienced patients, and the educational presentations were all good.” Respondent #32: “Talking with the mentor and watching the educational videos.”		All 55 respondents on the mentee satisfaction survey rated their overall experience as “good” (9/55, 16.4) or “excellent” (46/55, 83.6). 53/54 (98%) respondents selected “somewhat agree” or “strongly agree” when asked if they would recommend the Dialysafe peer-mentoring program to someone else in a similar situation.	
	Mentors	Mentor survey: “What did you like best about the Dialysafe program?” Respondent #14: “I loved being able to share my experience of dialysis to help others have a better dialysis experience.” Respondent #34: “I like the connection with other patients and for each party to know we are not alone in our situation.” Mentor survey: “How could we improve the Dialysafe program?” Respondent #29: “If the questions were tailored for each module, perhaps it would be more engaging for mentees and mentors.”		15 mentors completed 66 satisfaction surveys. On average, each mentor rated their experience with the Dialysafe peer-mentoring program as 1.4 (SD 0.5) on a 4-point Likert scale where 1=excellent. Mentors agreed that they would be willing to be a mentor in a program such as Dialysafe again (mean 1.2, SD 0.4; 1=strongly agree) and that they would recommend becoming a peer mentor for the Dialysafe program to a friend (mean 1.2, SD 0.4; 1=strongly agree).	
**Health goals**					
	Setting goals	Mentor survey: “Please explain why you would/would not want to be part of a similar program.” Respondent #20: “...the program assists patients in improving their knowledge and control over their health along with time to discuss/set goals with a mentor who has been in their shoes.”		52/54 (96.3%) mentees who completed the satisfaction survey felt that the Dialysafe program helped them set goals.	
	Reaching goals	“[Mentor] and I, through our ongoing discussions, she taught me and I learned how to control my fluids better. Before this, if I wanted something to drink I would get it and deal with it later... She taught me better fluid control and actually I do feel better.” (Patient representative, clinic N)		50/53 (94.3%) mentees who completed the satisfaction survey felt that the Dialysafe program helped them reach the goals they set.	
	Discussing goals with mentor	Mentee survey: “What did you like best about the Dialysafe program?” Respondent #17: “Getting to discuss problems and suggestions from mentor to solve problems.” Respondent #19: “Talking about ways to stay healthy.”		53/54 (98.1%) mentees who completed the satisfaction survey felt that it was helpful to discuss their goals with their mentor after each educational session.	
**Mentee-mentor relationship: My mentor was**...					
	Helpful	Patient representative: “I had a great mentor and we discussed a lot of things that we’ve talked about in the clinic. It really makes you feel better when you’re going through this dialysis to talk to someone who really understands.” (clinic N) Patient representative: “Well I thought it was really good that every week there were questions I had to answer and then when I talked with the gentleman we went over those questions and it helped to nail down what I should be doing and to be sure I’m doing that.” (clinic R)		53/55 (96%) mentees who completed the satisfaction survey agreed that their mentor was “strongly” or “somewhat” helpful.	
	Supportive	Mentee survey: “What did you like best about the Dialysafe program?” Respondent #33: “My mentor really listened to me & gave me good suggestions.” Respondent #34: “Having a great and understanding mentor!” Patient representative: “It really helped me, because when they first told me I had to do dialysis it was a depressing time for me at the beginning. When the study began, I got to talk to someone and it gave me a more positive attitude about what was going to happen and what will happen. I have a more positive attitude about being on dialysis.” (clinic L)		49/50 (98%) mentees who completed the satisfaction survey agreed that their mentor was “strongly” or “somewhat” supportive.	
	Knowledgeable	Mentee survey: “What did you like best about the Dialysafe program?” Respondent #10: “Talking to someone who’s been through what you’re going through exactly and having them be able to tell you about how different you feel after Transplant.” Respondent #40: “The information provided by my mentor.”		49/52 (94.2%) mentees who completed the satisfaction survey agreed that their mentor was “strongly” or “somewhat” knowledgeable.	
**Mentor experience**					
	The program allowed me to be helpful to my mentee(s)	Patient representative: “...I just thought it was good I had someone to talk to when I was starting dialysis and someone I could ask questions, and he gave really good answers.” (clinic R)		On average, the 15 mentors participating in peer mentoring rated the program’s opportunity to help their mentees as 1.3 (SD 0.5) on a 5-point Likert scale (1=strongly agree).	
	I had a good relationship with my mentee(s)	Mentor survey: “Please explain why you would/would not want to be part of a similar program.” Respondent #17: “It gives us both someone to talk to who understands what we’re going through.” Mentee survey: “How could we improve the Dialysafe program?” Respondent #52: “Let mentor & patient talk after the study is over...me & my mentor could be good friends.”		The 15 mentors participating in peer mentoring agreed that they had a good relationship with their mentee(s), with an average of 1.2 (SD 0.4) on a 5-point Likert scale (1=strongly agree).	
	My mentee(s) and I could trust each other	Patient representative: “It’s not the same as when you’re here [in the clinic] because everyone’s here, everyone can hear you talking. You feel more free to talk when it’s just the mentor. You can talk about fluids, exercise...” (clinic J)		The 15 mentors participating in peer mentoring agreed that there was mutual trust with their mentees, with an average of 1.2 (SD 0.2) on a 5-point Likert scale (1=strongly agree).	
	My mentee(s) made positive changes to their health because of our time together	Mentor survey: “Please explain why you would/would not want to be part of a similar program.” Respondent #58: “I would continue to be apart [sic] of this program because I can see how it’s helping the patients.” Mentor survey: “What did you like best about the Dialysafe program?” Respondent #38: “The ability to see the changes the mentees make and their health approve [sic].” Respondent #64: “...one man who was new on dialysis and almost a bit lost seemed to really benefit and make some good changes...I think he learned a lot from our conversations, made some great changes once he realized how to do things differently and liked the support of someone that had been there.”		The 15 mentors participating in peer mentoring felt that their mentees made positive changes to their health because of the time spent together, with an average of 1.8 (SD 0.6) on a 5-point Likert scale (1=strongly agree).	

#### Educational Content

The educational materials in each module were well received by mentees. The NKF educational video was particularly popular. The mentee survey #13 respondent said they liked “the videos and answering the questions after watching them,” and the mentee survey #14 respondent agreed that “the videos” were a highlight. The mentee survey #4 respondent said that, by completing the educational modules, they “found information I was unaware of during the sessions.”

#### Recommended Changes

Several mentees noted that the educational material was too basic for patients who had already had dialysis experience. The clinic B patient representative reflected, “More than likely it’s for beginners...I’m happy to be in the program, but there’s not much new information.” The mentee survey #53 respondent bluntly said: “Have beginner and advanced sessions. As a long time patient I learned very little.”

Another issue was the process of rescheduling mentoring sessions, either due to a missed session or an upcoming scheduling conflict. All sessions had to be rescheduled by a staff member (research team member from UMSI or NKF). One mentor (survey #32) suggested that the intervention could be improved by “making it easier to reach out to the patient. For example, I needed to change my meeting time I had to call NKF so they can reach out to the patient... maybe your system can have a simple limited chat for this situation.”

The challenging workflow around rescheduling mentoring sessions was exacerbated by the number of last-minute changes or missed sessions. The mentor survey #64 respondent explained, “It is very frustrating, however, the number of no-shows or last-minute cancelations. It gets to be a lot to sit at home, not get other things done or scheduled only to be left hanging.” Missed sessions ultimately impacted mentor satisfaction. Asked whether they would want to participate in similar interventions, the mentor survey #66 respondent said, “It would depend on whether the mentees were diligent in attending the sessions and following through.”

## Discussion

### Principal Findings

The Dialysafe intervention, offered to all eligible patients in the 10 clinics, enrolled 24.3% (155/639) of eligible patients. Over 50% (79/155) of mentees who initially agreed to participate left the intervention before their first mentoring session, and less than 10% (58/639) of eligible patients in Dialysafe completed the digital patient activation intervention. However, mentees who participated in at least 1 mentoring session left the intervention infrequently and were both highly engaged with the intervention and satisfied with the mentoring. Mentee engagement with the educational modules was low: 27.7% (43/155) to 52.9% (82/155) of mentees viewed or interacted with different parts of the content at least once. Among this group of participants, “communication with care team” was the most frequently changed health behavior according to self-reports; this finding was confirmed by staff in several clinics. Furthermore, over half of the mentees who completed the intervention reported that they had discussed what they learned during the intervention with one or more peers (32/55, 58.2%). Nearly two-thirds of these mentees also shared the intervention’s information sheet with friends and/or family (38/55, 69.1%).

### Equity in Recruitment, Engagement, Completion, and Attrition

A large proportion of eligible patients were not recruited into the intervention, and a small proportion finished it. This aligns with work on peer mentoring, which found that only 5% of hemodialysis patients who were eligible for peer mentoring enrolled in and started the program [72]. The likelihood of recruitment to the intervention was impacted by whether patients answered their phones (almost one-third did not), patients’ interest in peer mentoring, and practical considerations (eg, time constraints). Mentees who were recruited may represent the most highly activated patients at each clinic. Previous work has shown that 10% of patients receiving hemodialysis can be categorized as highly activated [73], which aligns with 9.1% of patients across all clinics completing the intervention. In adjusted analyses, mentees who were recruited into the intervention were younger, more educated, and had fewer comorbidities—all characteristics known to correlate with patient activation [74].

After being recruited, 50.3% (78/155) of participants did not actually start the digital patient activation intervention even though 89.7% (139/155) of them received this study’s tablet with loaded intervention content. Low participation after recruitment is in line with prior work on peer mentorship for chronic diseases, and among mHealth (mobile health) apps in general [54,75-78]; however, it is notable that those who managed to start the intervention typically did finish it. This finding could represent a “protective” effect of the mentor-mentee peer relationship formed. Additionally, the intervention offered tablets, training, and technical support that may have prevented attrition due to technical challenges once people started the intervention [59]. A small number of mentees completed portions of the educational modules yet never met with a mentor. These participants may have been interested in the digital patient activation intervention but either could not continue or could have lost interest after completing the educational materials and/or waiting for their finalized mentoring schedule. Only 72 of 639 (11.3%) eligible patients met with a mentor. Thus, despite high engagement among those who started it, this intervention will have limited clinic-wide impact unless reach and initiation improve. Wider implementation would require clinic-based outreach, rapid mentor matching, flexible scheduling, and extensive preimplementation testing of integrations among system components. As an integration problem delayed the intervention in the first 4 clinics, early attrition should not be attributed solely to lack of patient interest. Once mentees started the intervention, a different picture emerged. While age continued to be negatively associated with completing all mentoring sessions, it was not significantly associated with overall engagement scores. Instead, mentees who used a catheter for access or whose race was unknown had lower engagement rates. However, Black/African American mentees, a group that experiences health care disparities in numerous settings, also had significantly higher engagement. Notably, prior reviews have shown that disadvantaged populations [79], including female [80] and Black/African American [81] patients, may benefit more, on average, from peer-based interventions than their more advantaged, male, and White counterparts. Those with more comorbidities, especially anemia, also had higher rates of engagement. Taken together, these findings align with other work showing that the correlates of recruitment, engagement, and attrition in digital interventions differ [82]. However, these findings also complicate prior work on equity in engagement, showing that sicker and Black/African American patients engaged more once they joined the intervention. Notably, however, Black/African American hemodialysis patients do have consistently better longer-term outcomes on dialysis than their White counterparts [83]. They may also be less susceptible to IDH due to having higher baseline blood pressures than White patients [84].

Several features of the intervention were designed to reduce barriers to participation for patients with limited technology experience. Specifically, all mentees received a free tablet with the intervention preloaded, along with standardized onboarding, technology training, and ongoing technical support, as described previously [59]. Nevertheless, our eligibility criteria and engagement patterns point to concrete directions for improving equity in future versions of this intervention or similar others. The first consideration is language accessibility. In Dialysafe, the patient information sheet was available in Spanish, but peer mentoring and the educational materials were offered only in English, and patients who understood neither English nor Spanish could not be included in outcome monitoring. In future work, there would be value in recruiting and supporting bilingual mentors and providing educational materials in high-demand languages including Spanish. A second consideration is disability. Although the intervention technology underwent usability evaluation with hemodialysis patients during its development [58] and the tablets offered accessibility settings which were proactively enabled, these measures may not have been sufficient for all users. Moreover, accessibility is not always fully within the control of intervention designers, who must often integrate third-party platforms with their own accessibility features and constraints. Hence, future iterations could weigh accessibility support in platform selection and extend design processes and usability testing to focus specifically on patients with low vision and limited manual dexterity. Third, enabling the intervention to run on patients’ own devices could lower barriers for those reluctant to adopt an unfamiliar device, which we posit may make participation more comfortable for those with less technology experience. Such flexibility would complicate the provision of standardized technical support, however. Fourth, offering flexibility to participate in mentoring sessions at the dialysis clinic as an alternative to participation from home could extend the intervention’s reach to patients with limited privacy or environmental control at home. Nursing home residents, for example, proved difficult to recruit despite available procedures for reaching out to nursing home staff to facilitate their participation. Intervention participation while dialyzing might enable such patients to participate—although this option would need to address the difficulty of holding personal conversations in a busy clinic environment [85]. Fifth, tailoring educational content to a patient’s baseline knowledge could advance equity by reducing effort and time demands for the intervention, particularly for patients with lower health literacy or less technology experience. This leads to a sixth and final point: offering shorter interventions could accommodate time poverty and restricted control over one’s time, which often affect lower-income and disabled people [86]. Hemodialysis patients already devote substantial time to treatment, and briefer intervention components may make participation feasible for patients whose remaining time is limited or not fully theirs to schedule.

### Social Diffusion of Patient Activation Intervention Content

Many mentees reported sharing the information sheet for family members. This finding is important because family support can boost self-management for patients with ESKD [87], which can promote improved health [88-90]. Perhaps the information sheet provided an opportunity for interpersonal expressions of family support, particularly given that mentees who completed the satisfaction survey reported receiving strong family support for reaching their goals. That mentees shared the information sheet suggests that this low-burden approach could be adopted in other interventions for patients with complex conditions.

Slightly fewer mentees discussed the intervention with others in their clinic compared with their family/friend support system. While some mentees reported talking to others in their clinic during each treatment, mentees were more reserved or may have felt that holding a patient-to-patient conversation was difficult in a busy clinic setting [85]. Although most mentees did not report hearing about the intervention from other patients, 11 survey respondents did, suggesting that a small number of highly activated patients may contribute to conversations and knowledge sharing among peers. Future interventions could attempt to leverage patient word-of-mouth for recruitment and outreach to a greater degree than was possible during this time-limited study. Perhaps mentees who completed the intervention at an early stage could become intervention “ambassadors,” or be trained to participate as local clinic peer mentors, at later stages of a clinic’s intervention rollout.

### Reported Behavior Changes Stressed Patient-Care Team Communication

For mentee involvement in IDH prevention*,* the most frequently completed activity from the educational modules was goal setting, followed by passive viewing of the educational video at each module’s start. Goals under the “communication with care team” category were the most common; that category’s frequency highlights its importance to patients.

Hemodialysis patients vary in terms of whether, how much, how, and when they seek information about their health [91,92]. While health care providers are valued information sources, hemodialysis patients may face barriers to communication with their providers, including time limitations, perceived power differences between providers and patients, and health care experiences over their lifespan leading to a perception that the care team cannot be trusted [93-95]. Notably, dialysis facility staff in digital patient activation intervention clinics noticed and welcomed changes in patient conversations, including data sharing from self-tracking and clearly articulated questions and concerns about fluid management and IDH. Those who trust their health care providers rely on them more for health information, asking questions and remaining receptive to their advice [91]; we speculate that the intervention could have had some unmeasured impact on patient trust in providers.

Some mentees described health behavior changes in dialysis and fluid adherence—a finding supported by comments from mentors and clinic managers about their mentees. Empathetic support for peer mentoring has been shown to promote goal setting and self-management among predialysis patients and patients with ESKD [29,96]. Given the relatively small sample size, reports of patient behavior changes from mentees, clinic managers, and mentors are promising. However, because the recruited population in the digital patient activation intervention was self-selecting, this group of mentees may have been more capable of adopting positive behavior change.

Taken together, these findings converge on patient-care team communication as a pathway of benefit. Communication with the care team was the most frequently selected goal category in the modules (64/79, 81%, mentees) and the most common self-reported behavior change on the satisfaction survey (22/54, 40.7%, respondents). Staff in several clinics noticed patients asking clearer questions and sharing symptoms and self-tracked data between sessions. Communication was addressed in module 5, and mentees’ goals were revisited with mentors at each mentoring session. Each component of the intervention (education, goal setting, and peer mentoring) appeared to direct patient activation toward communication with the care team. Given known barriers to patient-provider communication in hemodialysis settings [93-95], we posit that facilitating this communication is a plausible mechanism by which such interventions could produce clinical benefit. As this convergence rests on self-reported and informally observed changes, it should be tested in future work using objective measures of communication and clinical outcomes.

Some of the Dialysafe study mentees felt that the intervention’s educational components were not challenging enough for long-term patients. Education tailored to patients is an important part of ESKD care and management, and patients often have limited knowledge about the condition [97-100]. Offering peer-mentoring sessions to patients new to dialysis or to patients preparing to change their treatment modality may facilitate tailoring educational topics to individuals at key treatment stages—an approach supported by patients [101]. Future interventions might take an alternative approach of tailoring the educational materials after assessment of baseline patient knowledge. Peer mentoring may be a way to promote individualized educational interventions and increase knowledge for new and experienced patients alike.

### Intervention Experience: Mentoring Relationships Were the Preferred Context for Education

In addition to the strong rapport facilitated by relational aspects of peer mentoring, knowledge sharing between mentors and mentees was well received. Mentors noted the value of sharing their hemodialysis experience with their mentees and the satisfaction of seeing their mentees make positive changes to their health. Several mentees reported learning from their mentor as a highlight, which aligns with prior work on the value of “experiential” information between patient peers, particularly practical strategies and personal stories. An oft-cited highlight of the digital patient activation intervention was the opportunity for mentees and mentors alike to talk to people in similar situations, because of the deeper understanding of lived experiences. Peer-based information includes attention to psychosocial issues related to living with illness, which may receive less focus in clinical settings [102].

Unfortunately, the number of mentee no-shows to scheduled mentoring sessions disrupted progress through the digital patient activation intervention. Mentee no-shows also affected overall mentor satisfaction. Although mentors understood the unique time commitment that dialysis therapy requires, frequent no-shows could disrupt the long-term sustainability of similar interventions. In future digital patient activation interventions using peer mentoring, it may help to allow mentees and mentors to reschedule their own meetings; however, intervention staff will almost certainly be needed to ensure that meetings progress as scheduled.

### Limitations

Several limitations to this study should be acknowledged. First, while the digital patient activation intervention was offered to all eligible patients, only a small proportion of eligible patients participated; thus, the group of recruits and participants was a self-selected one. These mentees may have been more engaged and more able to adopt or implement behavior change. Hence, these findings likely represent more motivated patients. Additionally, these mentees’ values, goals, and behaviors may not be representative of those prioritized by patients not participating in the intervention. Second, eligibility for participation in the digital patient activation intervention required English fluency; it was beyond this study’s scope to produce educational materials and recruit and train mentors in multiple languages. However, this requirement removed several otherwise eligible patients from the recruiting pool. Third, high patient discontinuation between recruitment and intervention start may have been partly linked to technical issues with the videoconference and PMMS integration, which delayed the intervention’s start for patients in the first 4 clinics. Later rounds of clinic and patient recruitment did not have this delay. Fourth, mentee satisfaction surveys were only completed by mentees who completed all 5 peer-mentoring sessions. Surveys were sent to those who completed 1-4 mentoring sessions, but no responses were received, and surveys were not sent to those who left the intervention before meeting with a mentor. As respondents chose to complete the full intervention, they were likely among its most engaged participants, and the high satisfaction reported here may overestimate satisfaction among all who started the intervention. The satisfaction findings should be interpreted with this selection in mind. A wider range of survey respondents would have provided more information on patients’ perceptions of the intervention’s strengths and weaknesses. Fifth, behavior change was assessed only through mentee self-report on the satisfaction survey and was not objectively verified against clinical or behavioral data. Self-reports are subject to social desirability and recall bias, and the survey was completed only by mentees who finished all 5 mentoring sessions, a group likely to view the intervention favorably. Clinic staff observations were consistent with some reported changes but were informal, and staff in other clinics did not observe such changes. Reported behavior changes should therefore be interpreted cautiously as preliminary signals rather than confirmed outcomes. Only the main behavior change was recorded, preventing analysis of whether mentees made multiple changes. Future evaluations should pair self-report with objective measures.

### Conclusions

Recruitment to the digital patient activation intervention was limited by the ability to reach eligible patients via phone call and by patient interest. After recruitment, most attrition was concentrated in the time preceding mentees' and mentors’ first mentoring session. While only a highly engaged subset of patients participated in the educational modules and peer-mentoring sessions, engagement scores and mentoring completion were high for those who did participate. Most mentees’ goals during the educational module completion component were related to improved communication with their care team. Likewise, health behaviors related to that goal were most often self-reported by mentees as their main behavior change. Interpatient communication about the intervention did occur, as did the sharing of an information sheet with family members. Hence, the social diffusion of information among mentees’ social networks represents an area for future study and potential intervention. Satisfaction surveys from mentees and mentors who completed the intervention reflect a high level of satisfaction, especially with the mentee-mentor relationship aspect. While some mentees indicated a preference for more advanced or in-depth information, even those who had several years of dialysis experience reported learning something new from the intervention. Indeed, both mentees and mentors reflected favorably on the ability to discuss health information with someone who had a similar life experience. Therefore, promoting disease-specific knowledge gain and health behavior change via relationships with a peer could be an avenue for future patient-centered research and quality improvement within hemodialysis clinics. In addition, the scalability and individualization of potential interventions should be considered, along with how to increase intervention reach to a greater proportion of patients at dialysis clinics.

## Appendix

Multimedia Appendix 1 Reasons for declining peer mentoring—joint display of qualitative and quantitative data.

Multimedia Appendix 2 Reasons for attrition from the patient activation intervention.

Multimedia Appendix 3 Mentee attrition from peer mentoring program based on completion of mentoring sessions.

Multimedia Appendix 4 Demographics of peer mentors.

Multimedia Appendix 5 Mentee use of intervention components: comprehension quizzes and goal setting modules.

Multimedia Appendix 6 Mentee interaction with videos in educational modules.

Multimedia Appendix 7 Percent of mentees engaging with intervention components.

Multimedia Appendix 8 Mentee perceived importance and confidence in behavior change of educational module topic (scale 1-10).

Multimedia Appendix 9 Mentees’ self-identified values. Mentees were asked to select their top 3-4 values during the first goal setting module.

Multimedia Appendix 10 Mentee goals identified in educational module.

Multimedia Appendix 11 Comprehension quiz scores.

## Abbreviations

CCI: Charlson Comorbidity Index
CKD: chronic kidney disease
EHR: electronic health record
ESKD: end-stage kidney disease
HIPAA: Health Insurance Portability and Accountability Act
IDH: intradialytic hypotension
IRR: incidence rate ratio
mHealth: mobile health
NKF: National Kidney Foundation
OR: odds ratio
PMMS: Peer Mentoring Management System

## Data Availability

The data underlying this paper cannot be shared publicly to preserve the privacy of individuals who participated in this study.
