# Supplementary material for: Recruitment, Engagement, Experiences, and Self-Reported Changes in a Digital Patient Activation Intervention to Improve Hemodialysis Session Stability: Mixed Methods Study
Source: J Med Internet Res. 2026 Oct 16;28:e93049. doi: 10.2196/93049 (PMC13722927; doi:10.2196/93049)
Supplement: Supplementary file 1 [file jmir_v28i1e93049_app10.pdf]

Module	Health Behavior Selected	n (%)
**1. Getting Enough Dialysis (n=77)**		
	Stay for all of my scheduled sessions	40 (52)
	Other (examples: talk to van service about transportation issues, talking to staff about home dialysis)	12 (15.6)
	Reduce the number of sessions I miss	6 (7.8)
	Try a new method for feeling more comfortable during dialysis (example: bringing pillows or a blanket)	5 (6.5)
	Write down my usual weight and usual weight gain between sessions (interdialytic weight gain, IDWG)	5 (6.5)
	Talk to a member of my dialysis team about a problem I'm having with staying for my full session	4 (5.2)
	Find an enjoyable new activity to do during dialysis	3 (3.9)
	Talk to a member of my dialysis team about a problem I'm having in getting to all my sessions	2 (2.6)
**2. Feeling Better with Less Salt/Sodium (n=71)**		
	Replace salt with another spice	14 (19.7)
	Other (example: mentee will cook their own meals instead of having housemates' meals)	12 (16.9)
	Talk to my dietician about how to limit salt/sodium in my diet	11 (15.5)
	Eat at home instead of going to a restaurant	10 (14.1)
	Choose low-sodium options at grocery store	7 (9.9)
	Read ingredients labels for food I buy	7 (9.9)
	Stop eating salty foods	5 (7)
	Keep a food diary that tracks my sodium intake	2 (2.8)
	Let the server know I want no extra salt in my food	2 (2.8)
	Make a new low-sodium meal	1 (1.4)
**3. Making Fluid Restrictions Work for You (n=66)**		
	Track the amount of fluid I drink	23 (34.9)
	Try a new method for controlling my thirst (example: lemon drops, frozen grapes, ice chips)	15 (22.7)
	Other (example: sip liquid from frozen drinks as they melt)	11 (16.7)
	Start the day with a pitcher of 32 ounces of fluid and drink out of it all day	6 (9.1)
	Become familiar with how much water is in the foods I eat	5 (7.6)
	Get smaller bowls and cups to use	3 (4.6)
	When I'm thirsty, do something that distracts me for 10-15 minutes before having something to drink	2 (3)
	Find a new, enjoyable distraction from drinking	1 (1.5)

	Ask my dialysis care team if I should schedule another dialysis session, or extend my time	0 (0)
	Ask a member of my dialysis team to show me where my total UF per hour is displayed on the dialysis machine	0 (0)
**4. Feeling Better on Dialysis and Having Easier Sessions (n=65)**		
	Tell a member of my dialysis team about a symptom I'm experiencing during or between dialysis sessions	34 (52.3)
	During my dialysis session, sit with my legs up and head back in my dialysis chair	6 (9.2)
	Track my blood pressure at home	6 (9.2)
	Track when I have IDH episodes, and remind my dialysis team about them	6 (9.2)
	Other (example: increase frequency of talking to dialysis center staff, take more frequent home blood pressures)	5 (7.7)
	Notify a member of my dialysis team that I've gained or lost weight	5 (7.7)
	Share my home blood pressure numbers with a member of my dialysis team	3 (4.6)
**5. Getting More Involved in Your Care (n=59)**		
	Share a concern about my health with a member of my dialysis team	22 (37.3)
	Other (example: selecting multiple goals)	11 (18.6)
	Record my symptoms, blood pressure, and weight using a tracking tool	8 (13.6)
	Ask a question or make a request about my treatment time or frequency	6 (10.2)
	Ask a question or make a request about my post-dialysis target weight	4 (6.8)
	Ask a question or make a request about my fluid removal speed (ultrafiltration rate)	4 (6.8)
	Make a list of all the medications I'm taking, including over-the-counter or herbal remedies	2 (3.4)
	Notify a member of my dialysis team that I've gained or lost weight	1 (1.7)
	Share an emotional concern with a member of my dialysis team	1 (1.7)
