# Supplementary material for: Recruitment, Engagement, Experiences, and Self-Reported Changes in a Digital Patient Activation Intervention to Improve Hemodialysis Session Stability: Mixed Methods Study
Source: J Med Internet Res. 2026 Oct 16;28:e93049. doi: 10.2196/93049 (PMC13722927; doi:10.2196/93049)
Supplement: Supplementary file 2 [file jmir_v28i1e93049_app11.pdf]

		Correct<br>n (%)	Incorrect<br>n (%)	No answer<br>n (%)
**Module 1 (n=59)**				
	Question 1	46 (78)	13 (22)	0 (0)
	Question 2	49 (83.1)	5 (8.5)	5 (8.5)
	Question 3	35 (59.3)	19 (32.2)	5 (8.5)
**Module 2 (n=51)**				
	Question 1	48 (94.1)	3 (5.9)	0 (0)
	Question 2	48 (94.1)	3 (5.9)	0 (0)
	Question 3	51 (100)	0 (0)	0 (0)
**Module 3 (n=50)**				
	Question 1	48 (96)	2 (4)	0 (0)
	Question 2	49 (98)	0 (0)	1 (2)
	Question 3	45 (90)	3 (6)	2 (4)
**Module 4 (n=49)**				
	Question 1	40 (81.6)	9 (18.4)	0 (0)
	Question 2	40 (81.6)	9 (18.4)	0 (0)
	Question 3	45 (91.8)	4 (8.2)	0 (0)
**Module 5 (n=46)**				
	Question 1	44 (95.7)	2 (4.3)	0 (0)
	Question 2	44 (95.7)	2 (4.3)	0 (0)
	Question 3	44 (95.7)	2 (4.3)	0 (0)
