# Supplementary material for: Recruitment, Engagement, Experiences, and Self-Reported Changes in a Digital Patient Activation Intervention to Improve Hemodialysis Session Stability: Mixed Methods Study
Source: J Med Internet Res. 2026 Oct 16;28:e93049. doi: 10.2196/93049 (PMC13722927; doi:10.2196/93049)
Supplement: Supplementary file 3 [file jmir_v28i1e93049_app3.pdf]

	Completed intervention (i.e., completed 5 peer mentoring sessions Mean (SD) or n (%) (n=58)	Attrition: Did not complete intervention (i.e., did not complete all 5 peer mentoring sessions Mean (SD) or n (%) (n=97)	P value[1]
**Age (years)**			
Age at time of enrollment	56.4 (13.8)	58.3 (13.9)	.41
**Sex**			
Male	40 (69.0)	47 (48.5)	.01*
**Race**			
Black/African American	21 (36.2)	39 (40.2)	.62
White	33 (56.9)	52 (53.6)	.69
Asian	0 (0)	1 (1.0)	.32
Native Hawaiian/Other Pacific Islander	0 (0)	1 (1.0)	.32
Other	2 (3.5)	1 (1.0)	.36
Unknown/missing	2 (3.5)	3 (3.1)	.90
**Ethnicity**			
Hispanic	6 (10.3)	13 (13.4)	.58
**Highest Education Achieved**			
Less than high school	3 (5.2)	8 (8.3)	.45
High school graduate or GED	18 (31.0)	37 (38.1)	.38
Some college or technical school	21 (36.2)	28 (28.9)	.34
College graduate	16 (27.6)	24 (24.7)	.70
**Clinical Characteristics**			
Charlson Comorbidity Index	3.4 (2.0)	3.0 (2.3)	.26
Sleep disorder	4 (7.0)	10 (10.3)	.50
Mental health disorder	7 (12.3)	20 (20.6)	.17
Anemia	48 (84.2)	76 (78.4)	.38
Dialysis vintage < 1 year	13 (22.4)	19 (19.6)	.68
Catheter access	9 (15.5)	19 (19.6)	.53

1. T-tests; categorical variables based on proportion of yes/no binomial.

*P<.05
