# Supplementary material for: Recruitment, Engagement, Experiences, and Self-Reported Changes in a Digital Patient Activation Intervention to Improve Hemodialysis Session Stability: Mixed Methods Study
Source: J Med Internet Res. 2026 Oct 16;28:e93049. doi: 10.2196/93049 (PMC13722927; doi:10.2196/93049)
Supplement: Supplementary file 4 [file jmir_v28i1e93049_app5.pdf]

	Comprehension Quizzes			Goal Setting Modules		
	**Completed 5 comprehension quizzes (n=46)**	**Completed 1-4 comprehension quizzes (n=18)**	**Completed 0 comprehension quizzes (n=91)**	**Completed 5 goal setting modules (n=58)**	**Completed 1-4 goal setting modules (n=21)**	**Completed 0 goal setting modules (n=76)**
**Age (years), mean (SD)**	53.9 (14.1)	55.8 (11.5)	59.8 (13.8)	55.3 (14.0)	59.7 (13.2)	58.8 (13.8)
**Sex, n (%)**						
Male	28 (60.9)	11 (61.1)	48 (52.7)	39 (67.2)	9 (42.9)	39 (51.3)
Female	18 (39.1)	7 (38.9)	43 (47.3)	19 (32.8)	12 (57.1)	37 (48.7)
**Race, n (%)**						
Black/African American	14 (30.4)	11 (61.1)	35 (38.5)	23 (39.7)	10 (47.6)	27 (35.5)
White	29 (63.0)	7 (38.9)	49 (53.8)	32 (55.2)	10 (47.6)	43 (56.6)
Asian	0 (0)	0 (0)	1 (1.1)	0 (0)	0 (0)	1 (1.3)
Native Hawaiian/Pacific Islander	0 (0)	0 (0)	1 (1.1)	0 (0)	0 (0)	1 (1.3)
Other	1 (2.2)	0 (0)	2 (2.2)	1 (1.7)	1 (4.8)	1 (1.3)
Unknown/missing	2 (4.3)	0 (0)	3 (3.3)	2 (3.4)	0 (0)	3 (3.9)
**Ethnicity, n (%)**						
Hispanic	5 (10.9)	1 (5.6)	13 (14.3)	6 (10.3)	3 (14.3)	10 (13.2)
Not Hispanic	38 (82.6)	15 (83.3)	71 (78.0)	48 (82.8)	17 (81.0)	59 (77.6)
Unknown	3 (6.5)	2 (11.1)	7 (7.7)	4 (6.9)	1 (4.8)	7 (9.2)
**Highest Education Achieved, n (%)**						
Less than high school	1 (2.2)	2 (11.1)	8 (8.8)	3 (5.2)	3 (14.3)	5 (6.6)
High school graduate or GED	15 (32.6)	4 (22.2)	36 (39.6)	16 (27.6)	9 (42.9)	30 (39.5)
Some college or technical school	16 (34.8)	5 (27.8)	17 (18.7)	20 (34.5)	2 (9.5)	16 (21.1)
College graduate	14 (30.4)	7 (38.9)	30 (33.0)	19 (32.8)	7 (33.3)	25 (32.9)
**Clinical Characteristics**						
Charlson Comorbidity Index, mean (SD)	3.0 (2.2)	2.7 (1.4)	3.3 (2.3)	3.0 (2.0)	3.4 (2.0)	3.2 (2.3)
Sleep disorder	2 (4.4)	1 (5.6)	11 (12.1)	3 (5.3)	2 (9.5)	9 (11.8)
Mental health disorder	7 (15.6)	4 (22.2)	16 (17.6)	7 (12.3)	7 (33.3)	13 (17.1)
Anemia	41 (91.1)	12 (66.7)	71 (78.0)	46 (80.7)	18 (85.7)	60 (78.9)
Dialysis vintage < 1 year	10 (21.7)	6 (33.3)	19 (20.9)	16 (27.6)	5 (23.8)	14 (18.4)
Catheter access	7 (15.2)	3 (16.7)	18 (19.8)	11 (19.0)	2 (9.5)	15 (19.7)

Note: Comorbidity data were missing for 1 participant; denominators for sleep disorder, mental health disorder, and anemia were 45, 18, 91, 57, 21, and 76 across the six columns, respectively.
