# Supplementary material for: Recruitment, Engagement, Experiences, and Self-Reported Changes in a Digital Patient Activation Intervention to Improve Hemodialysis Session Stability: Mixed Methods Study
Source: J Med Internet Res. 2026 Oct 16;28:e93049. doi: 10.2196/93049 (PMC13722927; doi:10.2196/93049)
Supplement: Supplementary file 5 [file jmir_v28i1e93049_app6.pdf]

	Educational Topic Video			Patient Story Videos		
	**Watched educational video from 5 modules (n=14)**	**Watched educational video from 1-4 modules (n=68)**	**Watched 0 educational videos (n=73)**	**Watched patient story video(s) from 5 modules (n=8)**	**Watched patient story video(s) from 1-4 modules (n=35)**	**Watched 0 patient story videos (n=112)**
**Age (years), mean (SD)**	57.6 (10.9)	56.0 (15.9)	59.1 (12.2)	57.4 (12.1)	57.8 (14.5)	57.5 (13.8)
Male	8 (57.1)	40 (58.8)	39 (53.4)	5 (62)	20 (57.1)	62 (55.4)
Female	6 (42.9)	28 (41.2)	34 (46.6)	3 (37.5)	15 (42.9)	50 (44.6)
Black/African American	5 (35.7)	28 (41.2)	27 (37)	3 (37.5)	17 (48.6)	40 (35.7)
White	9 (64.3)	35(51.5)	41(56.2)	4 (50)	16 (45.7)	65 (58)
Asian	0 (0)	0 (0)	1 (1.4)	0 (0)	0 (0)	1 (0.9)
Native Hawaiian/Pacific Islander	0 (0)	0 (0)	1 (1.4)	0 (0)	0 (0)	1 (0.9)
Other	0 (0)	2 (2.9)	1 (1.4)	0 (0)	2 (5.7)	1 (0.9)
Unknown/missing	0 (0)	3 (4.4)	2 (2.7)	1 (12.5)	0 (0)	4 (3.6)
Hispanic	2 (14.3)	7 (10.3)	10 (13.7)	0 (0)	3 (8.6)	16 (14.3)
Not Hispanic	12 (85.7)	54 (79.4)	58 (79.5)	6 (75)	31 (88.6)	87 (77.7)
Unknown	0 (0)	7 (10.3)	5 (6.9)	2 (25)	1 (2.9)	9 (8)
Less than high school	0 (0)	5 (7.4)	6 (8.2)	0 (0)	3 (8.6)	8 (7.1)
High school graduate or GED	3 (21.4)	22 (32.4)	30(41.1)	2 (25)	11 (31.4)	42 (37.5)
Some college or technical school	6 (42.9)	20 (29.4)	12 (16.4)	4 (50)	12 (34.3)	22 (19.6)
College graduate	5 (35.7)	21 (30.9)	25 (34.3)	2 (25)	9 (25.7)	40 (35.7)
**Clinical Characteristics**						

Charlson Comorbidity Index, mean (SD)	3.2 (1.5)	3.0 (2.2)	3.3 (2.2)	2.6 (2.1)	3.6 (2.2)	3.1 (2.2)
Sleep disorder	0 (0)	6 (8.8)	8 (11)	1 (14.3)	2 (5.7)	11 (9.8)
Mental health disorder	1 (7.7)	11 (16.2)	15 (20.6)	0 (0)	4 (11.4)	23 (20.5)
Anemia	11 (84.6)	55 (80.9)	58 (79.5)	7 (100)	29 (82.9)	88 (78.6)
Dialysis vintage < 1 year	2 (14.3)	20 (29.4)	13 (17.8)	3 (37.5)	12 (34.3)	20 (17.9)
Catheter access	3 (21.4)	9 (13.2)	16 (21.9)	1 (12.5)	6 (17.1)	21 (18.8)
