# Supplementary material for: Recruitment, Engagement, Experiences, and Self-Reported Changes in a Digital Patient Activation Intervention to Improve Hemodialysis Session Stability: Mixed Methods Study
Source: J Med Internet Res. 2026 Oct 16;28:e93049. doi: 10.2196/93049 (PMC13722927; doi:10.2196/93049)
Supplement: Supplementary file 6 [file jmir_v28i1e93049_app9.docx]

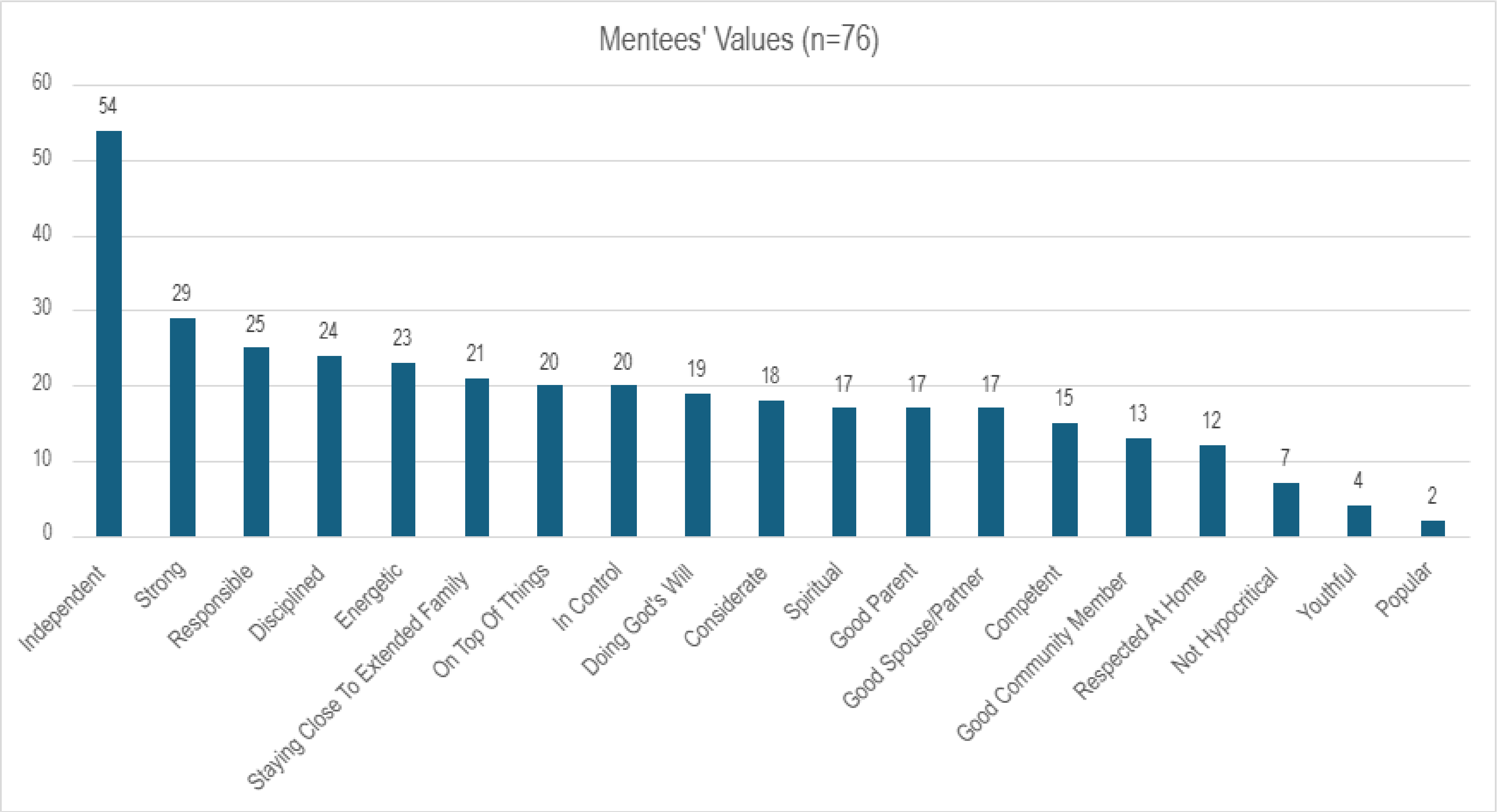

Mentees' Values (n=76)
60
50
40
30
20
10
0
54
29
25
24
23
21
20
20
19
18
17
17
17
15
13
12
7
4
2
Independent
Strong
Responsible
Disciplined
Energetic
Staying Close To Extended Family
On Top Of Things
In Control
Doing God's Will
Considerate
Spiritual
Good Parent
Good Spouse/Partner
Competent
Good Community Member
Respected At Home
Not Hypocritical
Youthful
Popular
