# Supplementary material for: Recruitment, Engagement, Experiences, and Self-Reported Changes in a Digital Patient Activation Intervention to Improve Hemodialysis Session Stability: Mixed Methods Study
Source: J Med Internet Res. 2026 Oct 16;28:e93049. doi: 10.2196/93049 (PMC13722927; doi:10.2196/93049)
Supplement: Supplementary file 6 [file jmir_v28i1e93049_app1.docx]

| **Reasons for Declining Peer Mentoring** | **Supportive Qualitative Data** | **Supportive Quantitative Data, n/N (%)** |
| --- | --- | --- |
| **Health Issues** |  |  |
| Eligible patients whose poor health prevented them from committing to participation in the patient activation intervention. | Clinic manager: *“No trends in refusal beyond people being elderly or in nursing homes*.” (Clinic F)  Clinic manager: “*It’s just a select few who wouldn’t do it for a million dollars. Most are engaged; they’re just aging.”* (Clinic D)  Patient representative: *“I’ll have to think about [becoming a mentor], I’ve had some health issues come up at home*.” (Clinic D)  A patient representative has run a support group for kidney patients’ families in the area since 2006, but *“there’s been high turnover due to the mortality rate…”* (Clinic B) | 30/272 (11) eligible patients declined to participate in the patient activation intervention due to health issues. |
| **Not comfortable in conversational English** |  |  |
| Eligible patients who did not feel confident in meeting with an English-speaking mentor and/or completing the educational modules in English. | Clinic manager: “*It’s a mix of languages [in the clinic]…we have some Hmong, Vietnamese, Cambodian…”* (Clinic T) | 41/272 (15) eligible patients declined to participate in the patient activation intervention because they were not confident in speaking/understanding the English-language education and mentoring sessions. |
| **No reason provided** |  |  |
| Eligible patients who declined participation in the patient activation intervention without providing a reason. | N/A | 36/272 (13) eligible patients declined to participate in the patient activation intervention without providing a reason. |
| **Not comfortable using technology** |  |  |
| Eligible patients who felt the technical component of the patient activation intervention would be too difficult and did not believe the technical support offered would be sufficient. | Clinic manager: *“I would predict that about 25% of patients, maybe more, will decline based on their comfort with technology*.” (Clinic F) | 31/272 (12) eligible patients declined to participate in the patient activation intervention because they did not want to use the technical component (tablet computer provided, email address required for account setup). |
| **Not interested in mentoring** |  |  |
| Eligible patients who did not feel the patient activation intervention would benefit them or felt that their health and fluid was already managed as well as possible. | *“At some point, the patients have to feel bad enough to make them WANT to feel better.”* (staff training session, Clinic C)  Clinic manager: *“…you can pretty much tell the patients who will say no right away. We have some, with our population…some pretty stern patients who don’t like to do anything extra.”* (Clinic H) | 90/272 (33) eligible patients declined to participate in the patient activation intervention because they did not feel that peer mentoring and/or the educational modules would improve their health. |
| **Too busy** |  |  |
| Eligible patients who did not feel they could commit to participating in the patient activation intervention due to existing commitments at work, school, and/or home. | Clinic manager: Patients see it as “*just another thing in their life.”* They have “*really complicated lives, more than you realize…their time at dialysis is just time to tune out, and a lot of them do.”* “*I wish more people would want to [join peer mentoring] but sometimes getting here is all they can do*.” (Clinic J)  “*[You] never know what’s going on in someone’s household…we have some older patients who are involved in helping out with grandkids.”* (staff training session, Clinic D) | 17/272 (6) eligible patients declined to participate in the patient activation intervention due to existing commitments. |
| **Eligibility issue** |  |  |
| Patients who knew that their eligibility would change within the next two months due to leaving their dialysis clinic or changing treatment modality. | N/A | 8/272 (3) eligible patients declined to participate in the patient activation intervention because they knew their eligibility would be changing in the near future. |
| **Dissatisfaction with other issues** |  |  |
| Eligible patients who declined participation in the patient activation intervention due to dissatisfaction or distrust in research, their existing care plan, etc. | Study champion: “*Some patients are excited about improving treatment for future patients, some more educated patients know about research studies, some think it’s a conspiracy for Fresenius to get information even though I told them there’s no identifying info.”* (Clinic C)  Clinic manager: “*I think COVID made it worse. Even bloodwork…some patients don’t want it checked weekly. They say, ‘I know you’re banking my blood!’ But we’ve had studies here before, and with RRI it isn’t foreign. I think that helps a little bit.”* (Clinic J)  Patient representative: *“We do have some weird patients, some moody patients. Some don’t want to be bothered. Some are rude. So when [CLINIC MANAGER] go around and ask those questions they think she’s up to no good… I think they’re just old and set in their ways. They don’t like change. Won’t answer the questions.”* (Clinic O) | 19/272 (7) eligible patients declined to participate in the patient activation intervention due to concerns outside the scope of the study. |
