# Supplementary material for: Recruitment, Engagement, Experiences, and Self-Reported Changes in a Digital Patient Activation Intervention to Improve Hemodialysis Session Stability: Mixed Methods Study
Source: J Med Internet Res. 2026 Oct 16;28:e93049. doi: 10.2196/93049 (PMC13722927; doi:10.2196/93049)
Supplement: Supplementary file 7 [file jmir_v28i1e93049_app2.docx]

| **Attrition Reasons** | | | | |
| --- | --- | --- | --- | --- |
|  | **Patient Request** | **Unable to Contact** | **No Longer Eligible** | **Health Reasons** |
| Eligible  (n=639) | 272 | 212 | 0 | 0 |
| Recruited  (n=155) | 4 | 7 | 4 | 1 |
| Sent Tablet  (n=139) | 14 | 20 | 4 | 4 |
| Completed/Opted Out of Technology Training  (n=97) | 4 | 1 | 2 | 1 |
| Matched  (n=89) | 4 | 3 | 2 | 3 |
| Scheduled  (n=77) | 0 | 1 | 3 | 1 |
| Session 1  (n=72) | 1 | 2 | 0 | 1 |
| Session 2  (n=68) | 0 | 3 | 0 | 4 |
| Session 3  (n=61) | 0 | 1 | 0 | 0 |
| Session 4  (n=60) | 0 | 1 | 0 | 1 |
| Session 5  (n=58) | Completed Peer Mentoring Program | | | |
