# Supplementary material for: Recruitment, Engagement, Experiences, and Self-Reported Changes in a Digital Patient Activation Intervention to Improve Hemodialysis Session Stability: Mixed Methods Study
Source: J Med Internet Res. 2026 Oct 16;28:e93049. doi: 10.2196/93049 (PMC13722927; doi:10.2196/93049)
Supplement: Supplementary file 8 [file jmir_v28i1e93049_app4.docx]

|  | **Mentors meeting with at least one mentee (n=15)** | **All mentors (n=26)** |
| --- | --- | --- |
|  |  |  |
| **Age (years), mean (SD)** |  |  |
|  | 52.4 (12.8) (n=14) | 50.7 (12.3) (n=21) |
| **Sex, n (%)** |  |  |
| Male | 5 (33.3) | 6 (23.1) |
| Female | 10 (66.7) | 16 (61.5) |
| Unknown | 0 (0) | 4 (15.4) |
| **Race, n (%)** |  |  |
| Black/African American | 7 (46.7) | 9 (34.6) |
| White | 7 (46.7) | 12 (46.2) |
| Asian | 1 (6.7) | 1 (3.9) |
| Unknown | 0 (0) | 4 (15.4) |
| **Ethnicity, n (%)** |  |  |
| Hispanic | 2 (13.3) | 2 (7.7) |
| Not Hispanic | 13 (86.7) | 20 (76.9) |
| Unknown | 0 (0) | 4 (15.4) |
| **Highest Education Achieved, n (%)** |  |  |
| Less than high school | 0 (0) | 0 (0) |
| High school graduate or GED | 1 (6.7) | 2 (7.7) |
| Some college or technical school | 5 (33.3) | 5 (19.2) |
| College graduate | 9 (60) | 19 (73.1) |
