# Supplementary material for: Recruitment, Engagement, Experiences, and Self-Reported Changes in a Digital Patient Activation Intervention to Improve Hemodialysis Session Stability: Mixed Methods Study
Source: J Med Internet Res. 2026 Oct 16;28:e93049. doi: 10.2196/93049 (PMC13722927; doi:10.2196/93049)
Supplement: Supplementary file 9 [file jmir_v28i1e93049_app7.docx]

| **Mentee Peer Mentoring Session Completion** | **% (n) of mentees who viewed educational video (n=82)** | | | | | **% (n) of mentees who completed comprehension quiz (n=64)** | | | | | **% (n) of mentees who completed goal setting (n=79)** | | | | |
| --- | --- | --- | --- | --- | --- | --- | --- | --- | --- | --- | --- | --- | --- | --- | --- |
|  | **Module 1** | **Module 2** | **Module 3** | **Module 4** | **Module 5** | **Module 1** | **Module 2** | **Module 3** | **Module 4** | **Module 5** | **Module 1** | **Module 2** | **Module 3** | **Module 4** | **Module 5** |
| 0 sessions (n=83) | 20.5 (17) | 3.6 (3) | 0 (0) | 1.2 (1) | 0 (0) | 10.8 (9) | 8.4 (7) | 6 (5) | 6 (5) | 4.8 (4) | 10.8 (9) | 8.4 (7) | 4.8 (4) | 4.8 (4) | 4.8 (4) |
| 1 session (n=4) | 25 (1) | 25 (1) | 25 (1) | 0 (0) | 25 (1) | 25 (1) | 25 (1) | 25 (1) | 25 (1) | 25 (1) | 75 (3) | 0 (0) | 25 (1) | 25 (1) | 25 (1) |
| 2 sessions (n=7) | 85.7 (6) | 28.6 (2) | 28.6 (2) | 14.3 (1) | 14.3 (1) | 57.1 (4) | 42.9 (3) | 42.9 (3) | 14.3 (1) | 14.3 (1) | 85.7 (6) | 57.1 (4) | 28.6 (2) | 28.6 (2) | 14.3 (1) |
| 3 sessions (n=1) | 100 (1) | 0 (0) | 0 (0) | 0 (0) | 0 (0) | 0 (0) | 0 (0) | 0 (0) | 0 (0) | 0 (0) | 100 (1) | 100 (1) | 100 (1) | 0 (0) | 0 (0) |
| 4 sessions (n=2) | 100 (2) | 50 (1) | 50 (1) | 50 (1) | 50 (1) | 0 (0) | 0 (0) | 50 (1) | 0 (0) | 0 (0) | 100 (2) | 100 (2) | 100 (2) | 100 (2) | 50 (1) |
| 5 sessions (n=58) | 72.4 (42) | 60.3 (35) | 60.3 (35) | 69 (40) | 62.1 (36) | 69 (40) | 69 (40) | 65.5 (38) | 72.4 (42) | 69 (40) | 94.8 (55) | 94.8 (55) | 96.6 (56) | 94.8 (55) | 87.9 (51) |
