# Supplementary material for: Recruitment, Engagement, Experiences, and Self-Reported Changes in a Digital Patient Activation Intervention to Improve Hemodialysis Session Stability: Mixed Methods Study
Source: J Med Internet Res. 2026 Oct 16;28:e93049. doi: 10.2196/93049 (PMC13722927; doi:10.2196/93049)
Supplement: Supplementary file 10 [file jmir_v28i1e93049_app8.docx]

| **Educational Module** | **n** | **Perceived Importance** | | **Confidence in Behavior Change** | |
| --- | --- | --- | --- | --- | --- |
|  |  | **How important, on a scale from 1-10, is it for you…** | **Mean (SD)** | **How confident, on a scale from 1-10, are you that you can succeed at…** | **Mean (SD)** |
| 1. Getting enough dialysis | 78 | …to make sure you get enough dialysis? | 9.1 (2.5) | …getting enough dialysis? | 8.9 (2.3) |
| 2. Feeling better with less salt/sodium | 71 | …to limit your sodium (salt) intake? | 9.1 (2.1) | …limiting your sodium (salt) intake? | 8.7 (2.1) |
| 3. Making fluid restrictions work for you | 66 | …to make sure you limit your fluid intake? | 9.1 (2.4) | …limiting your fluid intake? | 8.5 (2.4) |
| 4. Feeling better on dialysis and having easier sessions | 65 | …to prevent intradialytic hypotension (IDH)? | 9.5 (1.6) | …taking action to prevent IDH? | 8.8 (2.0) |
| 5. Getting more involved in your care | 59 | …to make sure you get enough dialysis? | 9.9 (0.5) | …getting enough dialysis? | 9.5 (1.5) |
